# UV-C LED wavelength effects on inactivation kinetics, DNA damage and membrane integrity in drinking water indicator bacteria

**DOI:** 10.1038/s41598-026-44556-8

**Published:** 2026-04-03

**Authors:** João Sério, Carolina Santos, Maria Eduarda Martins, Ana Paula Marques, Carolina Feliciano, Mónica Serrano, Adriano O. Henriques, Maria Teresa Barreto Crespo, Vanessa Jorge Pereira

**Affiliations:** 1 iBET - Instituto de Biologia Experimental e Tecnológica, Apartado 12, 2781-901 Oeiras, Portugal; 2 Instituto de Tecnologia Química e Biológica António Xavier, Universidade Nova de Lisboa, Av. da República, Oeiras, 2780-157 Portugal; 3 Present Address: INIAV, IP - Instituto Nacional de Investigação Agrária e Veterinária - Pólo de Inovação Dois Portos, Quinta da Almoinha, 2565-191 Dois Portos, Portugal

**Keywords:** Water treatment, Disinfection, Ultraviolet light emitting diodes, Wavelengths, Water quality indicators, Biological techniques, Biotechnology, Environmental sciences, Microbiology

## Abstract

Ultraviolet (UV) light-emitting diodes (LEDs) have emerged as a promising technology for water disinfection, offering selectable wavelengths that enable more precise targeting of specific cellular components. This study evaluated the inactivation efficiency of water quality indicators with different cell morphologies and envelope organization, *Escherichia coli* (Gram-negative) and *Enterococcus faecium* (Gram-positive), including both environmental and culture collection strains, using UV-C LEDs that emit light at 255 nm, 260 nm, 265 nm, 270 nm, and 280 nm. Inactivation kinetics as well as post-exposure repair, under dark and light conditions, were evaluated. Fluorescence microscopy observations and cyclobutane pyrimidine dimer formation were analyzed to elucidate morphological changes and DNA damage. Within the tested range, 265 nm LEDs achieved the highest inactivation rates for *E. coli* at a given UV fluence, consistent with the DNA absorption maximum in the 260–270 nm region. In contrast, *E. faecium* showed similar inactivation between 260 and 270 nm. Nevertheless, all tested wavelengths demonstrated high efficacy, achieving up to 6-log inactivation of both culture collection and environmental strains at low UV fluences (below 7 mJ/cm²). *E. faecium* showed enhanced resilience at lower fluences for all the wavelengths tested. Fluorescence microscopy revealed that both membrane integrity and DNA structure were increasingly affected by higher UV fluences, although signs of DNA damage were more pronounced and detectable even at lower exposure levels. Additionally, none of the strains tested showed considerable photoreactivation or dark repair capability, implying that the induced DNA lesions were largely irreversible under our experimental conditions. This study shows the efficacy of UV-C LEDs for water disinfection across a range of wavelengths, particularly at 265 nm, highlighting the important contribution of DNA damage to bacterial inactivation, and emphasizing their applicability for the water industry.

**Supplementary Information:**

The online version contains supplementary material available at 10.1038/s41598-026-44556-8.

## Introduction

Access to clean and safe water is a fundamental human right and a foundation of public health. Microbial contamination of drinking water remains a major cause of waterborne disease worldwide, with a particularly heavy burden in low-income countries where lack of safe water, sanitation and hygiene still leads to millions of diarrheal cases and deaths each year^1,2^. Effective disinfection is therefore a critical step to ensure water safety.

Water disinfection can be achieved by UV light, chlorination, chloramines, ozone treatment, among other techniques^3^. UV radiation displays many benefits, such as preserving the odor and taste of the water, keeping water beneficial minerals, typically not leading to the generation of disinfection byproducts, low maintenance requirements, relatively fast treatment rates and no risk of overdosing^4–6^. The process efficiency depends on several factors including water turbidity, UV wavelength, applied UV fluence, and the target microorganism^7–9^.

Conventional UV disinfection has been performed using low-pressure mercury lamps emitting at 254 nm, which are highly effective against a wide range of waterborne pathogens^10,11^ but depend on mercury, raising regulatory and environmental concerns considering phase-out policies^12^. As a result, mercury-free technologies such as excimer lamps^13,14^ and UV LEDs are increasingly considered for water treatment applications.

UV light-emitting diodes (UV LEDs) have emerged as a promising alternative to traditional UV mercury lamps for water disinfection, effectively overcoming key limitations, offering climate-responsive solutions, and introducing enhanced functionalities. Their compact size allows for more efficient reactor designs, while their ability to emit a wide variety of wavelengths enables targeted disinfection of specific microorganisms. UV LEDs feature faster start-up time, the ability to turn on and off instantly with high frequencies and low power consumption, making them energy-efficient and compatible with photovoltaic power sources, reducing operational costs. Furthermore, they are mercury-free, eliminating the environmental toxicity risks associated with mercury disposal^8,9,15,16^. Recent comprehensive reviews have highlighted the rapid maturation of UV LED technologies for water and wastewater disinfection, emphasizing both the wavelength dependence of microbial inactivation and the need to understand how different water matrices and reactor designs affect performance^9,17.^

While low pressure mercury lamps emit monochromatic light at 254 nm, UV LEDs that emit across a wide range of UV-C wavelengths can be acquired. This flexibility allows the optimization of the germicidal efficiency by targeting the action spectra of specific microorganisms, which are ultimately influenced by their unique composition of proteins and nucleic acids^9,18^. A delicate tuning in wavelength can enhance disinfection performance and reduce the UV fluence required to achieve the same microbial log reduction^10^.

The primary mechanism by which UV-C light inactivates microorganisms involves the formation of cyclobutane pyrimidine dimers (CPDs), covalent bonds between adjacent thymine or cytosine bases that distort the DNA helix. These lesions prevent proper base pairing, effectively blocking DNA replication and transcription^19^. CPDs are the most abundant and biologically relevant form of UV-induced DNA damage. Although some metabolic processes may persist, the inability to replicate renders the organism non-viable^20^.

*E. coli* and *E. faecium* were selected as model Gram-negative and Gram-positive drinking-water indicators, respectively. Gram-positive bacteria possess a thicker peptidoglycan cell wall and lack an outer membrane, whereas Gram-negative cells have a thinner peptidoglycan layer surrounded by an additional outer membrane^21–23^. These structural differences, together with species-specific DNA repair capacities, have been associated with distinct stress responses and can influence UV susceptibility and post-irradiation dark repair or photoreactivation^24–26^.

UV LEDs operating within the 255–265 nm range often achieve effective microbial inactivation by targeting the absorption maximum of DNA, inducing the formation of cyclobutane pyrimidine dimers (CPDs) that compromise replication and transcription^16,27–31^. Other studies have confirmed that LEDs near 265 nm effectively inactivate *E. coli* and other bacterial indicators at relatively low UV fluences, including in more complex water matrices featuring natural organic matter (NOM), although NOM can affect the disinfection efficacy through both shielding and promotion of oxidative stress^20,30,32^. Although diodes emitting at 255 nm can similarly damage DNA, their implementation remains constrained by current production efficiencies favoring 265 nm designs^27,32^.

By contrast, UV-C LEDs emitting around 270–280 nm rely on different mechanisms to microbial inactivation, including the degradation of key enzymes involved in DNA repair (e.g., photolyase) and the disruption of aromatic amino acids essential for protein function^20,33–35^. This protein-directed mechanism can impair photoreactivation and dark repair processes, thereby limiting bacterial regrowth following UV exposure^30,33^. Moreover, UV LEDs in this range often offer superior wall-plug efficiencies, enabling energy savings in full-scale systems^9,36,37^.

Recently, Ishida et al.^31^ evaluated UV LED inactivation of ten bacterial species across 13 wavelengths (250–365 nm) using a standardized irradiation system and showed that wavelength-dependent bacterial inhibition peaks at 263–270 nm, strongly correlating with cyclobutane pyrimidine dimer formation and outperforming low-pressure mercury lamps at equivalent UV fluences. These results provide an updated, multi-species action spectrum for UV LED disinfection and reinforce the importance of carefully selecting wavelength when designing LED-based reactors.

As UV LED technology continues to mature, pilot and full-scale reactors for drinking-water and wastewater disinfection are beginning to be implemented^11,38–40^, emphasizing their growing relevance for real-world applications.

Previous studies using UV-C LEDs have shown wavelength-dependent inactivation and DNA damage, and recent multi-wavelength work has derived action spectra across several collection strains under highly controlled conditions e.g.^6,9,31,36^. Most UV LED studies have focused on final viability measurements (log reduction) under different wavelengths and fluences e.g.^41,42^, and a more limited subset has examined bulk DNA damage such as CPD formation or related UV-induced lesions e.g.^24,29^, with relatively few addressing post-irradiation repair (photoreactivation or dark repair) e.g.^20,24,29^, or quantitative changes in DNA organization at the single-cell level.

In this work, we address these gaps by comparing five UV-C LED wavelengths (255 nm, 260 nm, 265 nm, 270 nm and 280 nm) for the inactivation of *E. coli* and *E. faecium* used both as culture collection strains and as environmental isolates recovered from natural water matrices. Under defined UV fluence conditions, we quantify wavelength-dependent inactivation kinetics, photoreactivation and dark repair, and CPD formation in the same isolates. In parallel, we apply fluorescence microscopy to evaluate membrane integrity and DNA organization and introduce a skewness-based, single-cell analysis of DAPI-stained nucleoids as a quantitative metric of UV-induced DNA reorganization. This provides a mechanistic assessment of how different UV-C LED wavelengths damage key drinking-water indicator bacteria and of the limited capacity of these organisms to repair UV-induced lesions under light and dark incubation conditions.

## Materials and methods

### UV Inactivation experiments

Inactivation assays were conducted using two UV LED reactors (PearlLab Beam, AquiSense Technologies™, USA) equipped with LEDs emitting at specific wavelengths in a Class II biological safety cabinet. The first reactor contains three LEDs emitting at 255 nm and 265 nm, while the second reactor contains three LEDs emitting at 260 nm, 270 nm, and 280 nm (both units operate at a voltage of 12 V and were fed with a power supply of up to 15 A at 230 V). Inactivation experiments, performed in duplicate, assessed the disinfection potential of these LEDs (see **Figure ****S1** in supplementary information section) against two strains of *E. coli* and two strains of *E. faecium* spiked in phosphate-buffered saline (PBS) solutions. Environmental strains of both organisms were isolated from real surface water samples (Tagus River, Algés Beach), and *E. coli* K12 MG1655 (DSM 18039) and *E. faecium* (DSM 109923) from culture collections were included for comparative analysis. 100 mL of the spiked PBS solutions were divided in two: 50 mL of sample exposed to UV LED radiation, and 50 mL of non-irradiated controls maintained in the dark to confirm that observed reductions in bacterial concentrations were exclusively due to UV-C exposure. Samples exposed to radiation were held in a custom-made, double-walled glass dish (**Figure ****S1****b** in supplementary information section) through which 4 °C water was recirculated to refrigerate the sample; the recirculating water remained completely separated from the sample and did not contact with it. LEDs were switched off at predefined exposure times, and the timer was paused during each sample collection that took less than 30 s. To enable direct comparison with other UV LED systems and previously published studies, exposure durations were translated into the corresponding UV fluence values. A detailed description of the UV LED spectral characterization, irradiance measurements and fluence determination procedures is provided in the Supplementary Information (**Section S1**).

#### Inactivation experiments of *E. coli* and *E. faecium*

Two culture collection isolates, the Gram-negative *E. coli* K12 MG1655 (DSM 18039) and the Gram-positive *E. faecium* (DSM 109923), along with two environmental isolates of each species, were fortified in phosphate buffer solutions for the inactivation assays. The isolation and identification procedures for the environmental *E. coli* and *E. faecium* strains recovered from surface water samples are described in detail in the Supplementary Information (**Section S2**). These bacteria were selected due to their roles as water quality indicators and their distinct cell wall structures. Additionally, culture collection and environmental strains were tested to assess variations in disinfection efficacy, since prior exposure to stressors such as heat^43,44^, acid^43–45^, desiccation^45,46^ and sunlight^47–49^ have been reported to enhance the resistance of different organisms to UV-C irradiation. This phenomenon is particularly relevant for environmental microorganisms, which may develop increased UV tolerance through prolonged solar radiation exposure. This effect was reported by Pereira et al.^47^, when comparing the direct photolysis inactivation rate constants of the same yeast species isolated from surface water and groundwater. Indeed, inactivation rates can differ substantially between isolates obtained from surface water versus groundwater, as well as between those isolated from environmental matrices and those kept in culture collection^50,51^. This trait may result from various factors, including the physiological state of the microorganisms, strain diversity, DNA repair mechanisms, and particle association^51^.

*E. faecium* and *E. coli* were cultivated overnight at 37 °C in brain heart infusion broth and Luria Bertani medium, respectively. Following incubation, cultures were centrifuged (6000 × g for 15 min) and washed twice with sterile phosphate-buffered saline to remove residual media. Optical densities at 600 nm were subsequently adjusted to 0.5 for *E. faecium* and 0.4 for *E. coli* using sterile PBS, yielding initial concentrations of approximately 10⁸ CFU/mL. The resulting cell suspensions were then used in UV-C inactivation assays.

To evaluate potential differences in UV-C resistance linked to bacterial growth stage, additional experiments were conducted with environmental *E. coli* cultures in both exponential and stationary phases. The experimental procedures followed those described previously, with the modification of sampling cells during the exponential growth phase. Evaluating UV-C susceptibility across distinct growth phases is critical, since stationary-phase cells have been reported to exhibit enhanced resilience to UV stress, attributed to the upregulation of DNA repair pathways and activation of global stress responses^52,53^. Testing in both growth phases intended to capture potential variability in UV-C resistance linked to bacterial physiological state, which may impact disinfection outcomes.

Bacterial quantification in the inactivation assays was done by colony-forming unit (CFU) counts on tryptic soy agar (TSA) plates divided by the sample volume. Undiluted and diluted samples were inoculated into TSA plates in duplicate, using glass beads for even spreading. Sterility controls were conducted by inoculating 100 µL of PBS into TSA plates to confirm the sterility of the glass beads, PBS, and media used. The TSA Petri dishes were incubated overnight at 37 °C and the CFU/mL were counted.

#### Reactivation experiments of *E. coli* and *E. faecium*

The potential for photoreactivation and dark repair was evaluated using the environmental strains of *E. coli* and *E. faecium* following UV-C inactivation. After exposure to LEDs emitting at different wavelengths with a UV fluence of 14 mJ/cm², samples were immediately transferred to an incubator (New Brunswick Innova 42, Eppendorf SE, Germany) for DNA repair assessment.

Five LED strips (Aigostar, 6500 K) were mounted in parallel on a metal tray placed inside the incubator to test photoreactivation. The samples were placed under the tray at a distance of 23.5 cm. The spectral characterization of each reactivation LED was performed using a spectrometer (UPRtek PG100N) and is provided in Table S2 in supplementary information section (measurements acquired in a straight line below each LED, at the same distance as the water level in the inactivation samples – 23.5 cm). Photoreactivation samples were directly exposed to these five LEDs, while the dark repair samples were enclosed in a black box that prevented any radiation from reaching the sample. Dark controls were kept in the same incubator to ensure the same conditions: agitation at 120 RPM and constant temperature of 27 °C throughout the incubation period.

To assess changes in reactivation over time, samples were collected at 2 h and 18 h post-incubation for colony forming unit enumeration. Previous studies indicate that maximum photorepair in *E. coli* typically occurs within the first 2–3 h post-irradiation, with rates plateauing thereafter^54,55^. Similarly, incubation periods ranging from 3 to 9 h have been shown to capture both dark and photorepair dynamics in *E. coli* under various conditions^56^. Therefore, the 2 and 18-hour incubation periods not only fall within this recovery window but also accommodates practical considerations, such as overnight incubation. Bacterial quantification was performed by counting the CFUs on TSA plates and expressed as CFU per milliliter of sample. Bacterial quantification was performed by counting CFUs on TSA plates and expressing the results as CFU per milliliter of sample. The subsequent calculation of log reductions, inactivation rate constants and associated kinetic parameters is described in detail in the Supplementary Information (**Section S3**).

### Analysis of Inactivation Mechanisms

#### Morphological Alterations

Fluorescence microscopy analyses were performed to assess morphological alterations of *E. coli* and *E. faecium* after the inactivation experiments performed in PBS. Before and after the assays, 3 mL of sample were collected and centrifuged at 1503 × g for 2 min to obtain a sediment. The cell pellet was then combined with 1 µL of FM™ 4–64 red dye (N-(3-Triethylammoniumpropyl)−4-(6-(4-(Diethylamino)Phenyl)Hexatrienyl)Pyridinium Dibromide), a lipophilic membrane dye at 10 µg/mL, and 1 µL of DAPI (4’,6-diamidino-2-phenylindole), a DNA stain at 5 ng/mL. After a second centrifugation, the supernatant was removed and 4 µL were placed on pre-prepared glass slides. The slides coated with 800 µL of agarose dissolved in distilled water at a concentration of 17 g/L, served as a support for the samples. After sample deposition on the slides, they were immediately analyzed to avoid any type of time-related degradation.

In additional tests, bacterial viability was evaluated using the LIVE/DEAD™ BacLight™ Bacterial Viability Kit (Invitrogen), which combines SYTO9 (a green nucleic acid stain that penetrates all bacteria) and propidium iodide (PI), which enters only cells with compromised membranes. The viability assay was performed by mixing the cell pellet with 1 µL of each dye, following the manufacturer’s protocol.

Microscopy images were acquired using a Leica DM6000B upright microscope equipped with an Andor iXon 885 EMCCD camera and controlled with the MetaMorph V5.8 software. The images were acquired with a 100 × 1.4 NA immersion objective and a 1.6x optovar, the fluorescence filter sets TX2, DAPI, FITC and phase contrast optics.

Microscopy images were analyzed using Fiji^57^, an open-source image processing package.

#### Dimer formation

To assess UV-induced DNA damage after the inactivation and reactivation experiments, cyclobutane pyrimidine dimers formation was determined as described by McDonald et al.^58^.

The environmental strains of *E. coli* and *E. faecium* were exposed to a UV fluence of 14 mJ/cm^2^ using each of the wavelengths tested. The cyclobutane pyrimidine dimers were also quantified before exposure and in the dark control samples (samples kept in the dark during the inactivation experiments, as shown in **Figure ****S1** in supplementary information section).

Briefly, the DNA was extracted from the samples and dark controls using the DNeasy^®^ UltraClean^®^ Microbial Kit (Qiagen, USA). Then, DNA was quantified using the NanoDrop ND-1000 Spectrophotometer (Thermo Fisher Scientific, USA). The DNA samples were diluted to 4 µg/mL and an enzyme-linked immunosorbent assay (OxiSelectTM UV-Induced DNA Damage ELISA Kit, CPD Quantification, Cell Biolabs, Inc, USA) was used to quantify the presence of CPDs in the DNA samples by comparing its absorbance with the absorbance of a CPD-DNA standard calibration curve. In each assay, a calibration curve was prepared using a supplied CPD-DNA standard (Part No. 232203), following the OxiSelect™ kit instructions, to quantify the CPD concentrations of the different samples.

## Results and discussion

### Inactivation experiments with LEDs

#### Inactivation Experiments of *E. coli* and *E. faecium*

The inactivation kinetics of environmental strains of *E. coli* and *E. faecium* isolated from surface water were assessed following exposure to LED UV-C irradiation. LEDs emitting at 255 nm, 260 nm, 265 nm, 270 nm, and 280 nm were used to deliver different UV fluences (up to 14 mJ/cm^2^), and the disinfection efficacy was evaluated across two independent biological replicates per wavelength.

Preliminary assays were first performed to evaluate the potential influence of bacterial growth phase on UV-C susceptibility. Exposure of *E. coli* cultures harvested during exponential and stationary phases to 280 nm UV LEDs at a fluence of 4 mJ/cm² revealed a slight but noteworthy variation: after exposure of the exponential phase cultures a small number of viable colonies were detected (average 19 CFU), whereas after exposure of the stationary phase cultures no detectable colony-forming units were counted (< 1 CFU).

Previous studies, however, reported increased UV-C resistance in stationary-phase *E. coli* cells^52,53^. This divergence may reflect strain-specific differences in stress adaptation and/or membrane properties, highlighting the variability of UV-C susceptibility among environmental isolates. Given the small difference observed, the wavelength-dependent inactivation experiments were conducted exclusively with stationary-phase cells.

**Fig. 1:**
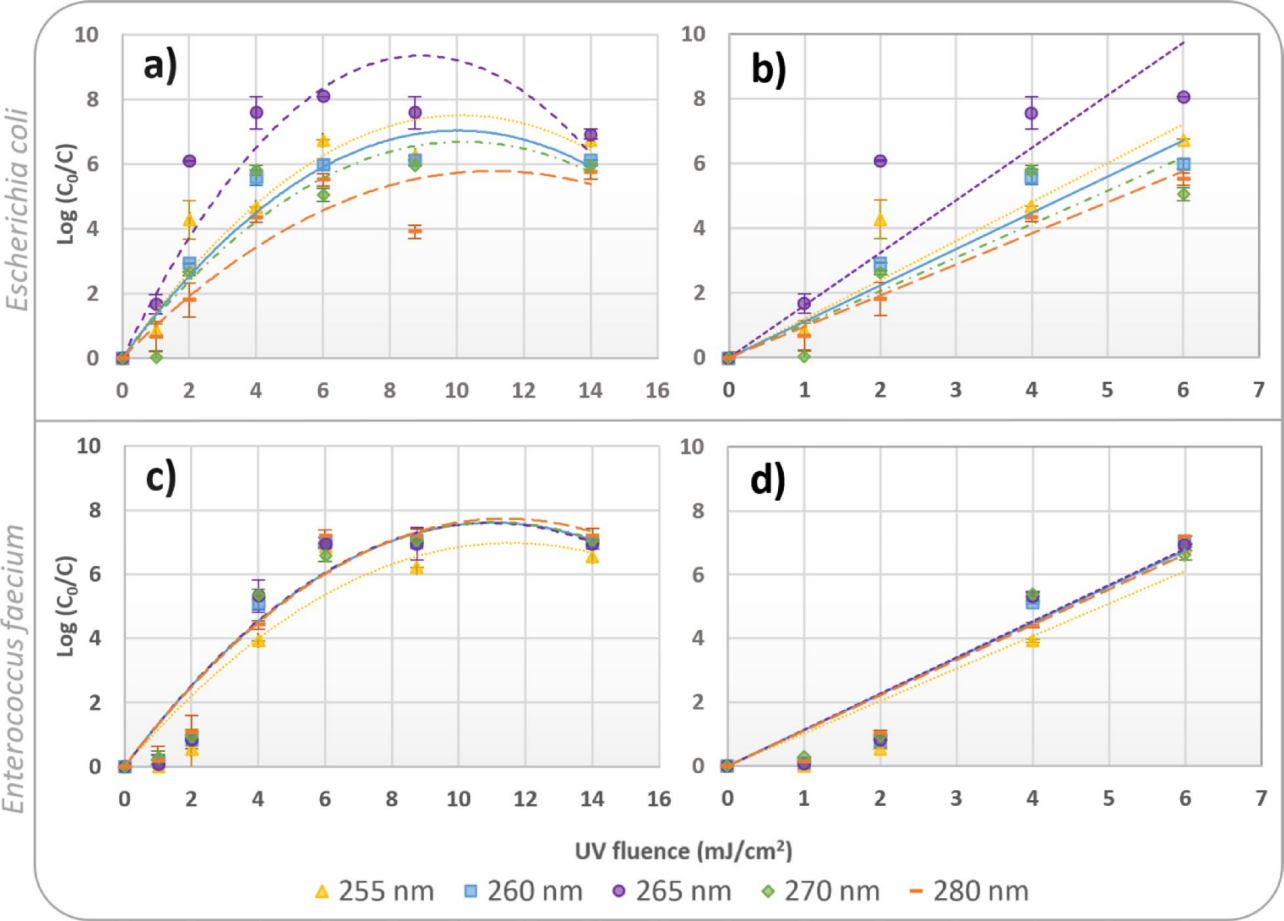
Inactivation of the environmental strains of *E. coli and E. faecium* [log (C_0_/C)] as a function of UV fluence using LEDs that emit light at different wavelengths: 255 nm, 260 nm, 265 nm, 270 nm, and 280 nm. (**a**) represents the polynomial regression of *E. coli* up to 14 mJ/cm^2^, while (**b**) shows the linear regression up to 6 mJ/cm^2^. (**c**) represents the polynomial regression of *E. faecium* up to 14 mJ/cm^2^, while (**d**) shows the linear regression up to 6 mJ/cm^2^. The error bars represent the results obtained in duplicate experiments.

Figure 1 shows the inactivation results obtained with the environmental strains, highlighting the effectiveness of each wavelength in reducing viable counts of the target bacteria. Higher log-reduction values indicate greater inactivation efficiency. 

Under ideal conditions, microbial inactivation follows first-order kinetics, characterized by a linear or exponential reduction in viable cells with increasing UV fluence. In all the inactivation experiments conducted in this study, tailing-off curves were observed. These curves are frequently reported in disinfection studies and may result from factors including intrinsic resistance within a fraction of the population, shielding by matrix components such as particle association or cellular aggregation^51,59^.

Since this study was conducted with extremely high concentrations of specific strains spiked into PBS, the most likely explanation for the observed phenomenon is cellular aggregation. Aggregated cells can create localized shielding effects, where inner cells are partially protected from UV exposure despite the overall homogeneity of the suspension^59^. Although the bacterial concentration used in this study exceeds those typically reported in real water matrices, similar shielding effects may also occur in natural systems due to the presence of more UV-resistant species or due to shielding by organic matter and suspended particles present in the water matrix^30^. To fit the experimental data over the entire fluence range (up to 14 mJ/cm²), a second-order polynomial regression was applied (Fig. 1a and c and Table S3 in supplementary information section), revealing the non-linear behavior associated with cell aggregation. A linear regression was also applied to the data obtained up to 6 mJ/cm² to compare the initial direct photolysis inactivation rate constants obtained using different wavelengths (Fig. 1b and d; Table 1).

Regarding the linear regression analysis, the inactivation efficiency of the various wavelengths differed among the target bacteria. For *E. coli*, the LEDs emitting at 265 nm achieved higher inactivation rates compared to 270 nm and 280 nm, with over a 2-log reduction difference after a UV fluence of 6 mJ/cm². In contrast, *E. faecium* demonstrated a similar inactivation efficiency across all wavelengths.

A detailed examination of varying UV fluences revealed that *E. faecium* was more resilient up to 2 mJ/cm², especially after exposure to 255 nm and 265 nm, showing only about 1-log inactivation, whereas in the assays spiked with *E. coli* about 6-log inactivation were measured at the same UV fluence using the LEDs that emit light at 265 nm. However, the susceptibility of *E. faecium* increased substantially at 4 mJ/cm², achieving nearly 5-log inactivation. From 4 mJ/cm² to 6 mJ/cm², *E. faecium* inactivation kept increasing consistently.

The enhanced UV resistance of *E. faecium* may be attributed to structural and physiological characteristics of Gram-positive bacteria. Unlike Gram-negatives, these species possess a thick peptidoglycan cell wall that serves as a robust barrier, potentially limiting UV-C penetration and delaying damage to intracellular components. Jaiaue et al.^60^ demonstrated that Gram-positive bacteria, namely *Staphylococcus aureus* and *Bacillus subtilis*, require higher fluences for equivalent inactivation than *E. coli*, directly linking this resistance to cell wall structure. Additional studies have shown that peptidoglycan architecture contributes not only to envelope rigidity but also to membrane protein assembly and cellular stress resilience, further reinforcing its protective role^61,62^. Additionally, *Listeria monocytogenes* (Gram-positive) has been reported to possess more efficient DNA repair pathways, such as photoreactivation, SOS repair systems, and nucleotide excision repair systems^63^. *B. subtilis* has also shown greater UV resistance than *E. coli* in combined UV-chlorine disinfection, which the authors linked to more resilient cellular structures and possible enzymatic defenses^26^. Similarly, *S. aureus*, another Gram-positive bacterium, was found to exhibit a higher survival rate under UV-C exposure than *E. coli* and *Salmonella Typhimurium*, a difference attributed to its thicker peptidoglycan layer and associated envelope robustness^64^.

Table 1 summarizes the UV sensitivity of each microorganism across the different wavelengths, measured by the UV fluence based inactivation rate constant for direct photolysis, which corresponds to the slope of the linear regressions of the results presented in Fig. 1b and d.

**Table 1:** Fluence based inactivation rate constants (kf) obtained in duplicate experiments (and standard deviations) for the environmental strains of *E. coli* and *E. faecium* after exposure to LEDs that emit light at different wavelengths (255 nm, 260 nm, 265 nm, 270 nm, and 280 nm). The coefficient of determination of the linear regression (R^2^) is presented in square brackets.

LEDs wavelenght (nm)	E. coli	E. faecium
	kf (cm^2^/mJ) [*R*^2^]	kf (cm^2^/mJ) [*R*^2^]
255	1.202 ± 0.065 [0.955]	1.019 ± 0.002 [0.939]
260	1.120 ± 0.047 [0.949]	1.126 ± 0.001 [0.954]
265	**1.624 ± 0.056 [0.926]**	**1.136 ± 0.015 [0.951]**
270	1.030 ± 0.024 [0.917]	1.111 ± 0.026 [0.957]
280	0.959 ± 0.053 [0.992]	1.109 ± 0.012 [0.965]

The inactivation rate constants (*kf*) for both *E. coli* and *E. faecium* were similar across the tested wavelengths, with the highest values observed after exposure to 265 nm. This effect was more pronounced in the inactivation assays using the LEDs emitting light at 265 nm to inactivate the environmental strain of *E. coli*, with a kinetic constant of 1.624 ± 0.056 cm²/mJ—the highest observed across all wavelengths and strains tested. This result is in strong agreement with the absorption maximum of DNA and confirms the relevance of 265 nm LEDs for targeting nucleic acid damage^4,6,37,65–67^. Consistent with these results, previous studies have reported enhanced microbial inactivation efficiencies at shorter UV-C wavelengths, particularly around 255–265 nm, with increasingly reduced disinfection effectiveness as wavelength increases. Bowker et al.^68^ demonstrated high inactivation rates of *E. coli* ATCC 11229 at 255 nm using UV-C LEDs, while Rattanakul and Oguma^37^ observed that *E. coli*, *Pseudomonas aeruginosa* and *Legionella pneumophila *were more effectively inactivated at 265 nm than at longer wavelengths, with efficacy declining toward 280 nm under equivalent fluences. Additional studies further support the observed trend of diminished bacterial inactivation efficiency at longer UV-C wavelengths e.g.^6,35,67,69^. Beck et al.^6^ compared inactivation across multiple UV-C LED wavelengths using *E. coli*, *B. subtilis*, and MS2 bacteriophage, and found decreased performance at 280 nm, with peak efficacy near 260–265 nm. These results highlight the wavelength dependence of disinfection performance, which several works link to the action spectra of DNA damage, with Beck et al.^6^ discussing the correlation of inactivation efficiency with the absorption peak of nucleic acids. Moreover, Ishida et al.^31^ recently compared 13 UV LED wavelengths (250–365 nm) and ten bacterial species using a standardized irradiation protocol and found that inactivation consistently peaked at 263–270 nm, with log-reductions closely tracking CPD and 6–4 PP dimer production. Their deconvolution analysis identified maximal inhibition near 267.6 nm, further supporting the concept that the highest germicidal efficacy lies close to DNA absorption maximum.

In accordance with these findings, recent reviews of UV LED disinfection have discussed that bacterial inactivation rate constants typically peak between 254 and 270 nm, with *E. coli* often inactivated more efficiently at 260–265 nm than at 254 nm^9,17^.

The environmental strain of *E. faecium* demonstrated a consistent inactivation profile across the UV-C spectrum. In contrast to *E. coli*, its sensitivity was less wavelength-dependent, with kinetic constants oscillating narrowly from 1.019 ± 0.002 cm²/mJ to 1.136 ± 0.015 cm²/mJ between 255 nm and 265 nm. The LEDs emitting light at 265 nm also achieved a slightly higher inactivation rate constant of *E. faecium*, although the value was very similar to those obtained using the other tested wavelengths. This pattern, with *E. faecium* requiring slightly higher or similar fluences than *E. coli* to reach the same log reduction, is consistent with recent LED-based studies reporting lower sensitivity of Gram-positive species compared with Gram-negative bacteria under UV-C exposure^31,70^.

The inactivation results of culture collection strains of *E. coli* and *E. faecium* after using the LED reactors that emit light at different wavelengths are shown in Figure S4 (supplementary information section), while Table S4 (supplementary information section) summarizes the inactivation rate constants for both organisms. Similar inactivation rate constants were observed for the tested culture collection strains, regardless of the wavelength used and the strain tested. This observation is consistent with findings by Kamel et al.^69^, who reported that environmental strains of *E. coli* and *E. faecalis* isolated from wastewater showed equal to or higher than reported inactivation rates for ATCC strains under similar conditions. Similarly, a full-scale wastewater study by Silva et al.^71^ revealed that both clinical and environmental strains of *E. coli and Klebsiella* sp. exhibited comparable responses to UV disinfection, emphasizing that adaptive responses in natural environments do not guarantee increased UV resistance and that strain-to-strain variability occurs. Combinations of the different UV-C wavelengths were also tested, and no synergies were obtained compared to the disinfection with the individual wavelengths^72^. Using dual-LED configurations, Beck et al.^6^ has reported that combining wavelengths within the UV-C range has shown little benefit beyond a single optimal wavelength, which is consistent with the obtained results.

Figures 1 and S4 show that all tested wavelengths achieved high inactivation levels, exceeding 4-log reduction with relatively low UV fluences. The high coefficients of determination in Table 1 and Table S4 indicate that the linear regression explains well the results, and the inactivation constants can be used to estimate the necessary UV fluence to achieve different inactivation levels (Table 2). Table 2 shows that extremely similar UV fluences are needed to achieve a certain level of inactivation of the target environmental and culture collection strains, and that very low UV fluences (between 2 mJ/cm² and 5 mJ/cm²) are required for a 4-log reduction. Typical UV fluence levels applied for microbial inactivation range from approximately 40 to 186 mJ/cm², depending on target organisms and treatment objectives^73,74^. If LEDs are applied for water treatment, UV fluences higher than the values tested in this study will have to be applied to ensure the inactivation of more resistant pathogens, such as viruses and protozoa.

**Table 2:** UV fluence (in mJ/cm²) required to achieve different levels of inactivation (2-log, 4-log, and 6-log) for the target environmental and culture collection strains of *E. coli* and *E. faecium* after exposure to LEDs that emit light at different wavelengths (255 nm, 260 nm, 265 nm, 270 nm, and 280 nm).

			UV fluence (mJ/cm^2^)		
λ (nm)	Organism	Strain	2-log	4-log	6-log
255	*E. coli*	Environmental	1.7	3.3	5.0
		Culture collection	1.5	3.0	4.5
	*E. faecium*	Environmental	2.0	3.9	5.9
		Culture collection	2.2	4.4	6.7
260	*E. coli*	Environmental	1.8	3.6	5.4
		Culture collection	1.9	3.9	5.8
	*E. faecium*	Environmental	1.8	3.6	5.3
		Culture collection	2.2	4.4	6.5
265	*E. coli*	Environmental	1.2	2.5	3.7
		Culture collection	1.4	2.9	4.3
	*E. faecium*	Environmental	1.8	3.5	5.3
		Culture collection	2.4	4.7	7.1
270	*E. coli*	Environmental	1.9	3.9	5.8
		Culture collection	1.9	3.7	5.5
	*E. faecium*	Environmental	1.8	3.6	5.4
		Culture collection	1.6	3.2	4.8
280	*E. coli*	Environmental	2.1	4.2	6.3
		Culture collection	1.7	3.4	5.1
	*E. faecium*	Environmental	1.8	3.6	5.4
		Culture collection	1.8	3.7	5.5

#### Reactivation experiments

**Fig. 2:**
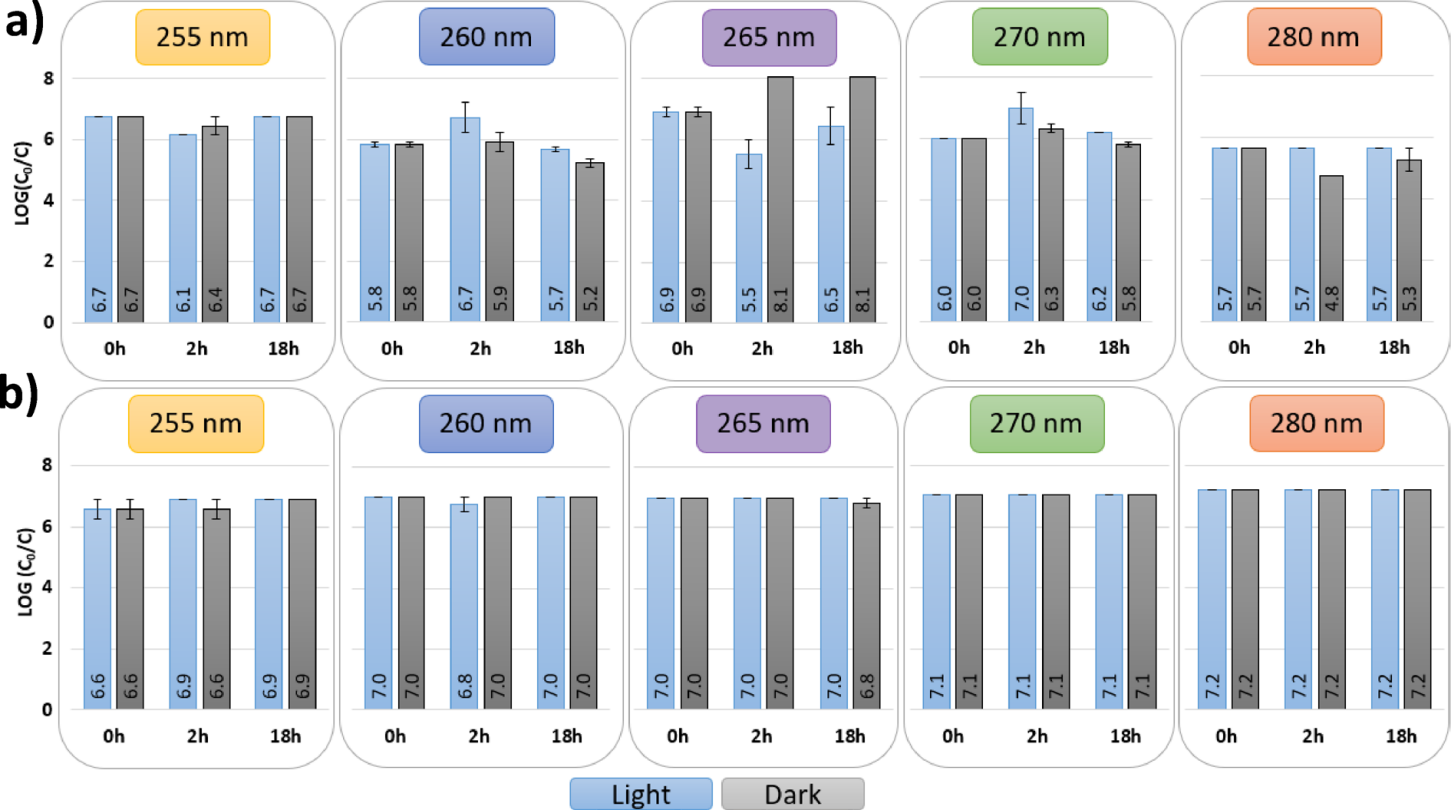
Logarithmic reduction of (**a**) *E. coli* (top) and (**b**) *E. faecium* (bottom) after exposure to LEDs emitting light at wavelengths of 255 nm, 260 nm, 265 nm, 270 nm, and 280 nm, with a UV fluence of 14 mJ/cm², immediately after exposure (0 h), after two (2 h) and eighteen hours (18 h) of treatment, in light (blue colored bars) and dark (grey colored bars) conditions. Error bars represent duplicate results.

Given that bacteria have demonstrated the ability to repair UV-induced DNA damage^75^, environmental strains of *E. coli* and *E. faecium* samples were tested after exposure in terms of their potential to reactivate following the inactivation experiments. Figure 2 shows the logarithmic reduction of these organisms immediately after exposure (0 h), as well as after 2 h and 18 h of incubation in both light and dark conditions.

In contrast to various studies reporting post-UV reactivation in *E. coli* and other bacterial species^54,76,77^, no substantial photoreactivation or dark repair was observed in the environmental isolates of *E. coli* and *E. faecium* tested in this work. After 18-hour incubation periods under both light and dark conditions, log-reduction levels remained mostly unchanged when compared to post-irradiation (0 h), indicating that no substantial recovery occurred under the tested conditions. This result was consistent across all wavelengths evaluated (255 nm, 260 nm, 265 nm, 270 nm and 280 nm), suggesting that neither wavelength nor light condition was sufficient to trigger a pronounced repair activity in these strains. This observation aligns with previous reports indicating that 280 nm UV exposure can suppress bacterial repair mechanisms, potentially through damage to photolyase or other repair-associated enzymes^20,33^. However, limited reactivation was observed at shorter wavelengths: at 260 nm, approximately 0.5 log recovery occurred under dark conditions after 18 h, and at 265 nm, a similar magnitude of photoreactivation (~ 0.46 log) was detected under light conditions. These findings are in line with previous reports of partial photoreactivation in *E. coli* following UV exposure at 260–265 nm, with recovery typically ranging from 0.5 log to 0.6 log^20,33^, though lower reactivation (~ 0.1 log) has also been observed under similar conditions^34^. Nonetheless, no substantial recovery was detected at all the wavelengths tested, despite the applied fluence (14 mJ/cm²) falling within the range previously associated with repair phenomena.

Several factors may influence the apparent absence of reactivation. One possibility is that these environmental isolates lack functional photorepair systems, such as photolyase, due to genetic loss, mutation, or downregulation. On the other hand, cells may have experienced irreversible damage during UV exposure, including membrane disruption or oxidation, which could suppress both photoreactivation and dark repair pathways. This interpretation aligns with work by Xiao et al.^78^ and Song et al.^79^, who demonstrated that additional environmental stressors, such as UV-A pre-irradiation or ROS generation, can selectively impair DNA repair mechanisms, particularly photoreactivation, which relies on a single enzyme and is more vulnerable to protein damage.

Moreover, the prolonged reactivation period (18 h) used here should, in theory, have allowed sufficient time for any repair-capable cells to restore viability, as supported by prior studies^34,54–56,80^. Therefore, the lack of substantial regrowth or recovery suggests a legitimate absence of effective repair rather than insufficient incubation time. This outcome questions the robustness of repair systems in environmental bacteria, indicating that UV-C disinfection targeting these strains may achieve more permanent inactivation than anticipated.

In addition to direct DNA repair mechanisms, population-level processes may also influence the survival and recovery of UV-damaged cells. A small subpopulation of phenotypically tolerant “persister” cells can survive and later reactivate, providing a transient survival strategy at the community level, although the molecular triggers of persistence remain only partially understood^81^. Intercellular communication via quorum sensing can upregulate general stress responses and increase bacterial tolerance to multiple environmental stresses when the local cell density is high^82,83^. In the context of UV treatment, recent work has shown that UV-induced viable-but-non-culturable *E. coli* displays limited and slow resuscitation in water with low nutrient content, whereas resuscitation is enhanced when cells are incubated in media with a high nutrient content in the presence of biofilm-derived extracellular polymers and quorum-sensing signals^84,85^. These findings suggest that persister cell formation and quorum sensing could, in principle, support long-term survival after UV exposure. However, such mechanisms are likely to be strongly constrained under the conditions used in our reactivation assays, where cells were incubated in PBS as free suspensions, without biofilm attachment or additional organic carbon sources.

In a real-scale application, where much higher UV fluences are applied, reactivation is unlikely to occur.

### Analysis of inactivation mechanisms

#### Morphological alterations

Fluorescence microscopy images were acquired to assess the morphological features (membrane and DNA damage) of *E. coli* and *E. faecium* before and after exposure to UV LEDs emitting at 255 nm, 260 nm, 265 nm, 270 nm, and 280 nm, using a fixed UV fluence of 14 mJ/cm². Both species were stained with FM4-64 and DAPI prior to irradiation and immediately after exposure, and representative images for each wavelength and organism are presented in the Supplementary Information (Figures S5–S8).

**Fig. 3:**
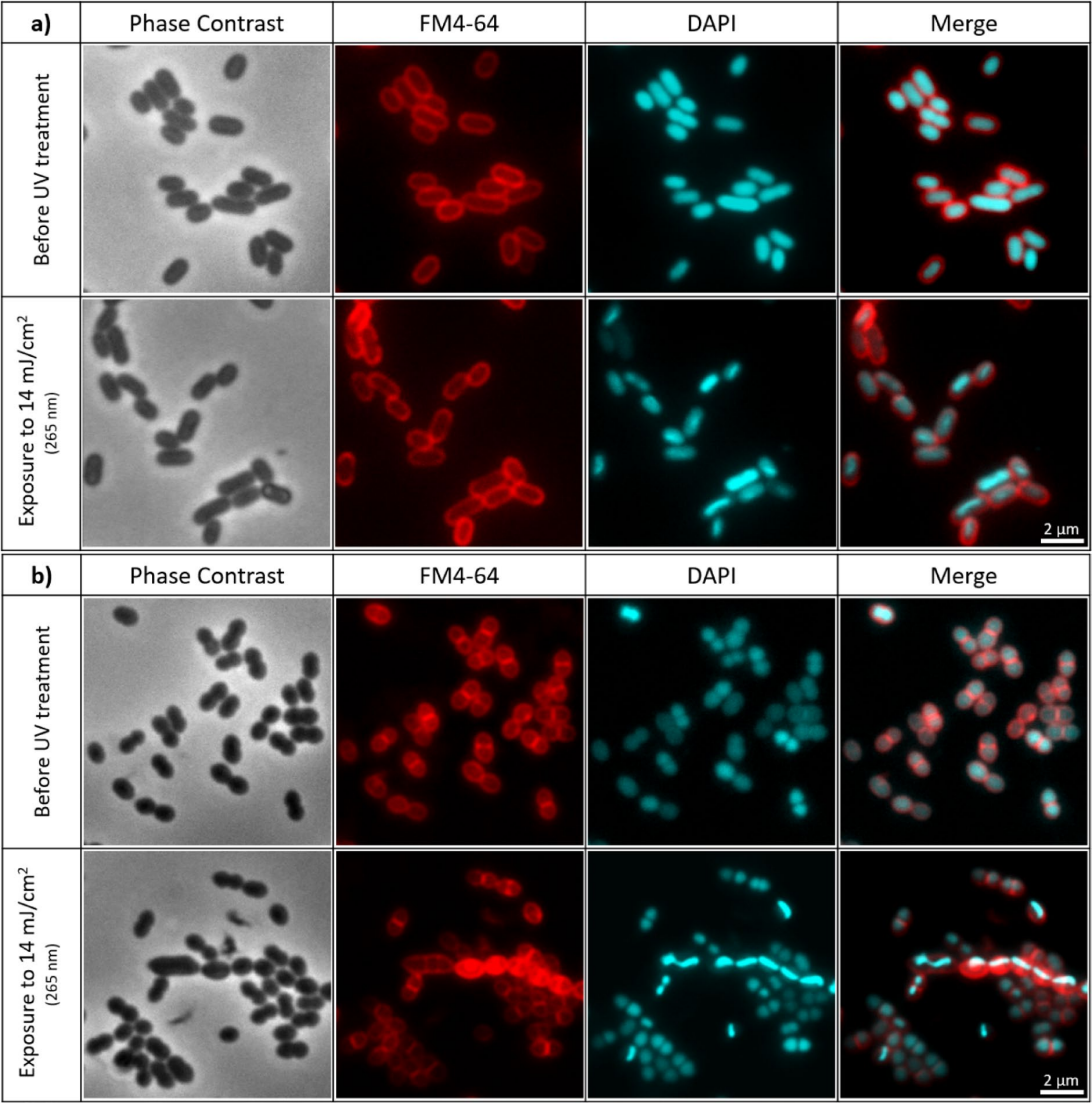
Fluorescence microscopy images illustrating membrane integrity and DNA organization in stationary-phase *E. coli* (**a**) and *E. faecium* (**b**) before and after UV-C LED exposure at 265 nm (UV fluence of 14 mJ/cm²).From the left side, the first column shows phase contrast microscopy images (grayscale), the second shows membrane staining with FM4-64 (red), the third displays DNA stained with DAPI (cyan), and the fourth shows the merged images of FM4-64 and DAPI. These images show that, under these conditions, most cells retain apparent membrane integrity while displaying UV-induced alterations in DAPI-stained nucleoids. A larger field of view for the same conditions is presented in Figure S9 in supplementary information section.

In the main text, we focus on 265 nm (Fig. 3 and Figure S9), which was the wavelength that yielded the highest inactivation rate for *E. coli* and is representative of the qualitative staining patterns observed at the other wavelengths. Since the inactivation experiments raised questions regarding membrane integrity and DNA morphological changes, additional complementary microscopy experiments were carried out using *E. coli*, including SYTO9/PI viability staining, a fluence series at 265 nm and a skewness-based single-cell analysis of DAPI intensity distributions.

**Fig. 4:**
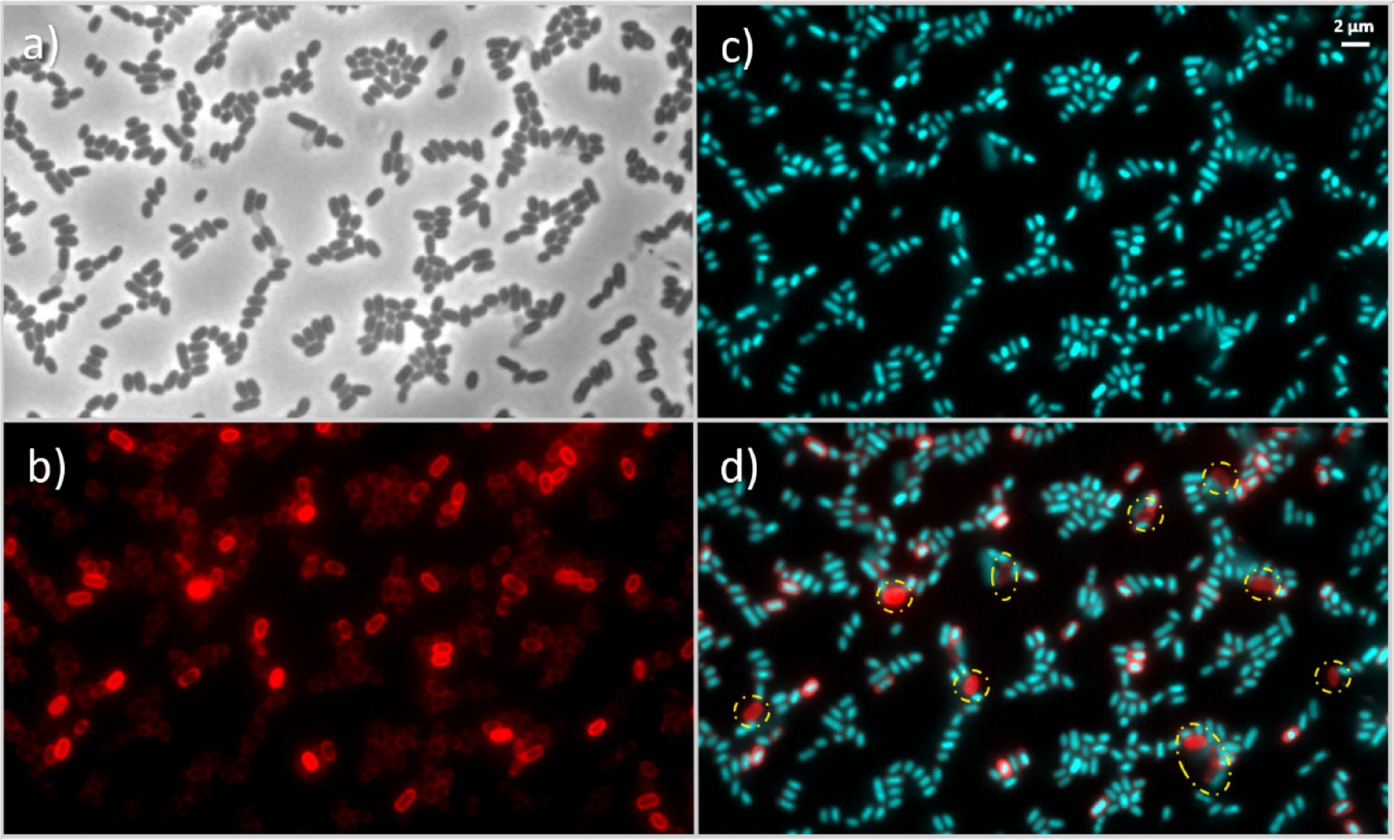
Example of stationary-phase *E. coli* cells retaining membrane staining but lacking DAPI signal after UV-C LED exposure at 265 nm at a UV fluence of 14 mJ/cm². Image **a**) shows the phase contrast, **b**) displays membrane staining with FM4-64 (red), **c**) shows DNA staining with DAPI (cyan) and **d**) the merge images of FM4-64 and DAPI channels. Yellow circles highlight cells with FM4-64 signal in the absence of corresponding DAPI fluorescence, illustrating cases in which membrane staining is retained while nuclear material is no longer detected by DAPI, consistent with severe perturbation of DNA structure or accessibility.

As shown in Fig. 3, FM4-64 staining (red channel) indicates that the cell membranes of *E. coli* and *E. faecium* appear mostly unaffected following exposure to UV-C LEDs, suggesting membrane integrity was preserved under these treatment conditions, with similar patterns being observed at the other wavelengths in the Supplementary Information section (Figures S5 and S7). However, a more detailed comparison with DAPI staining (Fig. 4) reveals anomalies in specific cells: while the FM4-64 signal is retained, these cells exhibit an absence of DAPI fluorescence, indicating that their DNA is no longer detected by this stain and is therefore likely to be severely damaged, displaced or inaccessible.

**Fig. 5:**
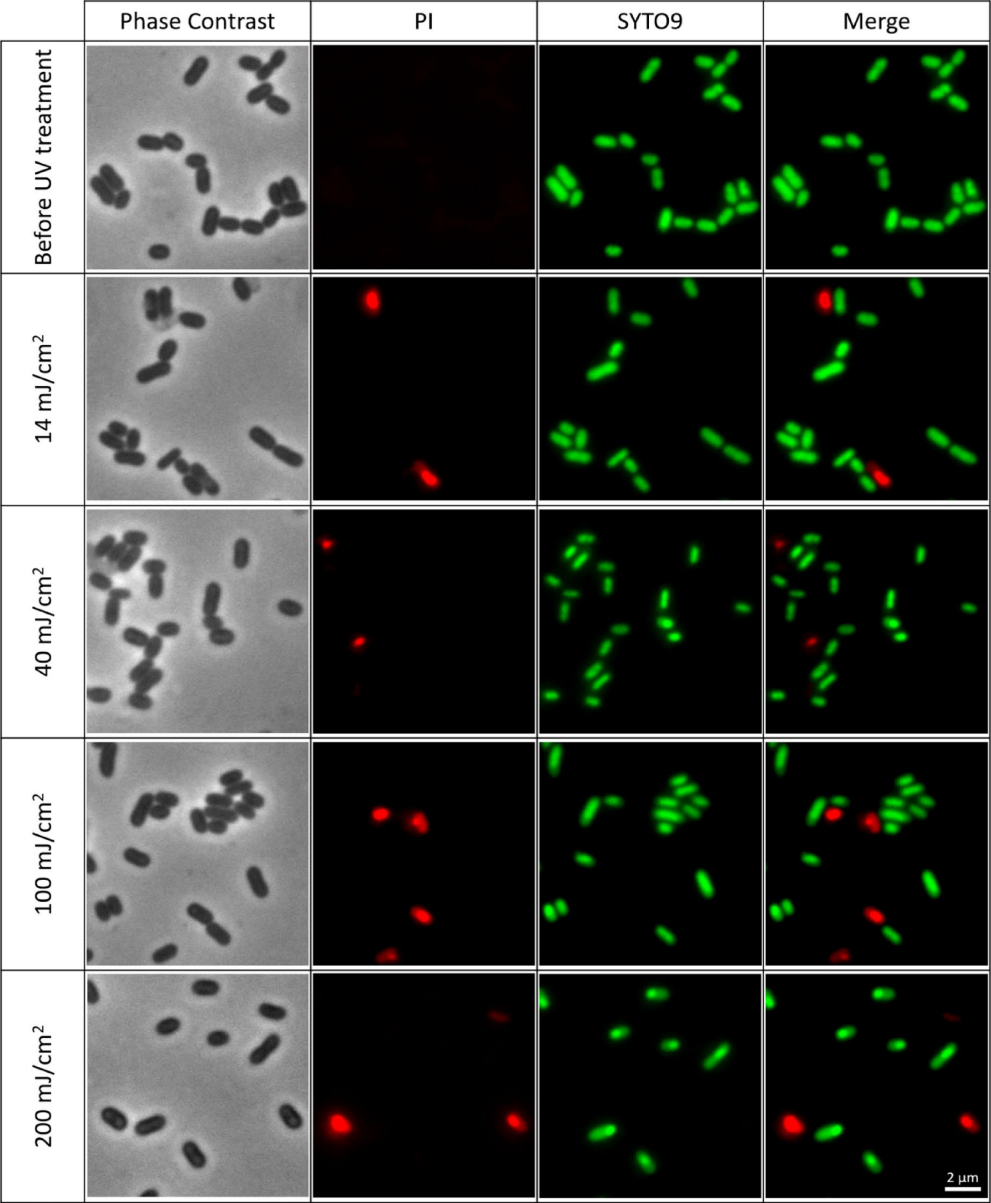
Fluence-dependent loss of membrane integrity in *E. coli* revealed by SYTO 9/propidium iodide staining after UV-C LED exposure at 265 nm at UV fluences of 14 mJ/cm², 40 mJ/cm², 100 mJ/cm², and 200 mJ/cm². From the left side, the first column presents phase contrast images (grayscale), second shows non-viable cells stained with propidium iodide (red), the third shows viable cells stained with SYTO9 (green), and the fourth shows the merged images of PI and SYTO9 channels. The images illustrate a progressive increase in PI-positive (membrane-compromised) cells with increasing fluence, while many cells remain SYTO9-positive. A larger field of view for the same conditions is presented in Figure S10 in supplementary information section.

This observation is further supported by complementary staining using SYTO9 and propidium iodide (PI) in additional experiments (using various UV fluences), which revealed PI-positive signals in a subset of cells – Fig. 5 and S10 (in Supplementary Information section). An increase in the number of PI-positive cells was observed with increasing UV fluence, indicating a higher fraction of cells with compromised membranes. To evaluate this trend, a quantitative analysis was performed by calculating the proportion of PI-positive cells from three microscopy images per UV fluence (**Table S5** in supporting information). On average, 407 cells were scored per image, with counts ranging from 102 cells to 702 cells depending on the field of view and cell density. The results showed an increase in PI-positive cells from an average of 0.8 ± 0.3% at 0 mJ/cm² to 2.9 ± 0.9%, 5.1 ± 1.3%, 5.5 ± 1.5%, and 5.7 ± 0.2% at 14 mJ/cm², 40 mJ/cm², 100 mJ/cm², and 200 mJ/cm², respectively, indicating a fluence-dependent increase in membrane-compromised cells following UV-C exposure.

Since PI only penetrates cells with damaged membranes, this staining serves as an indirect marker of membrane integrity loss following UV-C exposure. In these experiments, cells that became PI-positive lost their SYTO9 signal, indicating that SYTO9 fluorescence was quenched by PI, which is consistent with competitive binding of PI to nucleic acids in cells with damaged membranes. Although these cells retained a rod-shaped morphology under phase contrast, their fluorescence profile shifted from SYTO9-positive/PI-negative to SYTO9-negative/PI-positive. These observations are consistent with previous reports describing membrane damage and altered nucleic acid staining patterns in bacteria and yeasts exposed to UV-C LED irradiation^86,87^. However, our microscopy data only provides indirect evidence, and higher-resolution analyses would be needed to fully clarify this phenomenon.

Previous studies have described pronounced UV-induced morphological alterations in bacteria, including ghost-like cells, bleb protrusion, membrane vesiculation, cell lysis, and microcolony formation, often accompanied by visible deformation or collapse of the cell envelope, particularly at higher fluences or in surface-associated systems^88–92^. In contrast, under our experimental conditions (planktonic cells in PBS, 14–200 mJ/cm²), most *E. coli* and *E. faecium* retained a rod-shaped morphology in phase-contrast images, despite clear evidence of membrane permeabilization (PI staining) and nucleoid reorganization (DAPI). Only occasional small clusters were observed, without the extensive structural deformation described in other systems. These findings suggest that the UV damage detected here may correspond to an intermediate stage in which membrane integrity and DNA organization are affected while gross cellular morphology still remains preserved. Higher resolution analyses (e.g., electron microscopy) would be required to verify whether subtle envelope alterations are also present.

Regarding the DAPI staining, changes were also observed after the inactivation experiments. Although the genetic material appeared uniformly distributed prior to UV treatment, the fluorescence signal became uneven, with brighter and more condensed areas, following exposure—particularly at higher UV fluences. These findings point to possible DNA condensation. Vauclare et al.^93^ reported that UV radiation, particularly in the UV-C range, can induce nucleoid remodeling in radiation-resistant bacteria such as *Deinococcus radiodurans*, leading to DNA condensation. Similarly, Shibai et al.^94^ suggested that these less homogeneous regions in *E. coli* may signal cellular stress caused by UV exposure. The condensed staining patterns observed in *E. coli* and *E. faecium* after exposure to LEDs emitting at different wavelengths aligns with this UV-induced nucleoid condensation, which may reflect the bacterial DNA damage response (seen in Figure S6 for *E. coli* and Figure S8 for *E. faecium* in Supplementary Information section). However, no noticeable differences were detected between wavelengths. It is important to note that this condensation is usually a transient response, whose extent and duration varies depending on bacterial species, UV fluence, and environmental factors^93,94^. Nonetheless, in this study, the conformational changes persisted in the samples retrieved from the reactivation experiments, suggesting no reversion to the native DNA state (as shown in Figure S11 for *E. coli* and Figure S12 for *E. faecium*).

**Fig. 6:**
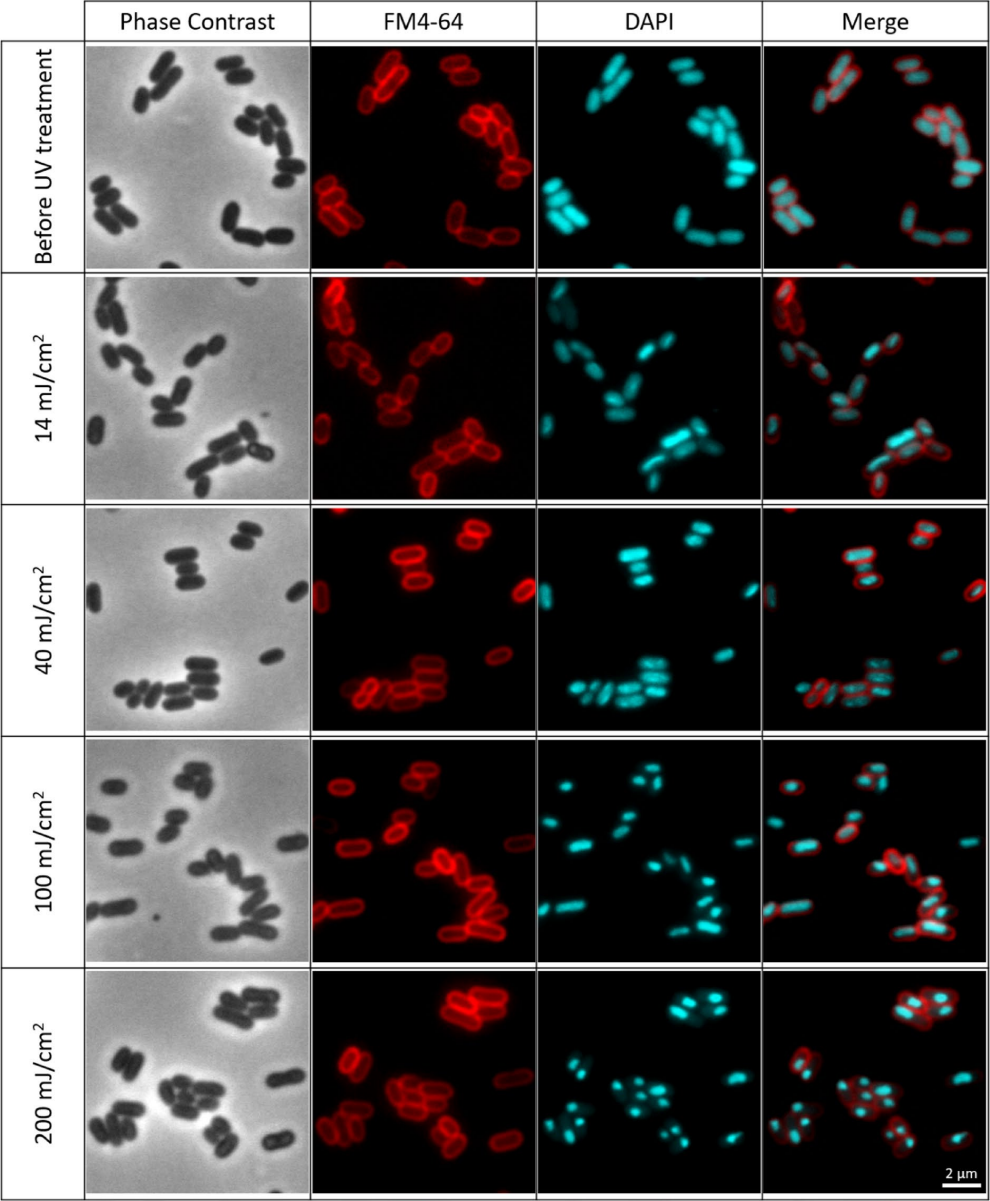
Fluence-dependent changes in DAPI-stained nucleoid organization in stationary-phase *E. coli* before and after exposure to 265 nm UV-C LEDs at fluences of 14 mJ/cm², 40 mJ/cm², 100 mJ/cm², and 200 mJ/cm². From the left side, the first column presents phase contrast images (grayscale), the second shows membrane staining with FM4-64 (red), the third displays DNA stained with DAPI (cyan), and the fourth shows the merged images of FM4-64 and DAPI. As UV fluence increases, progressive alteration in the morphology of the nucleoid becomes evident, transitioning from a homogeneous intracellular distribution to a more condensed and localized structure. A larger field of view for the same conditions is presented in Figure S13 in supplementary information section.

**Fig. 7:**
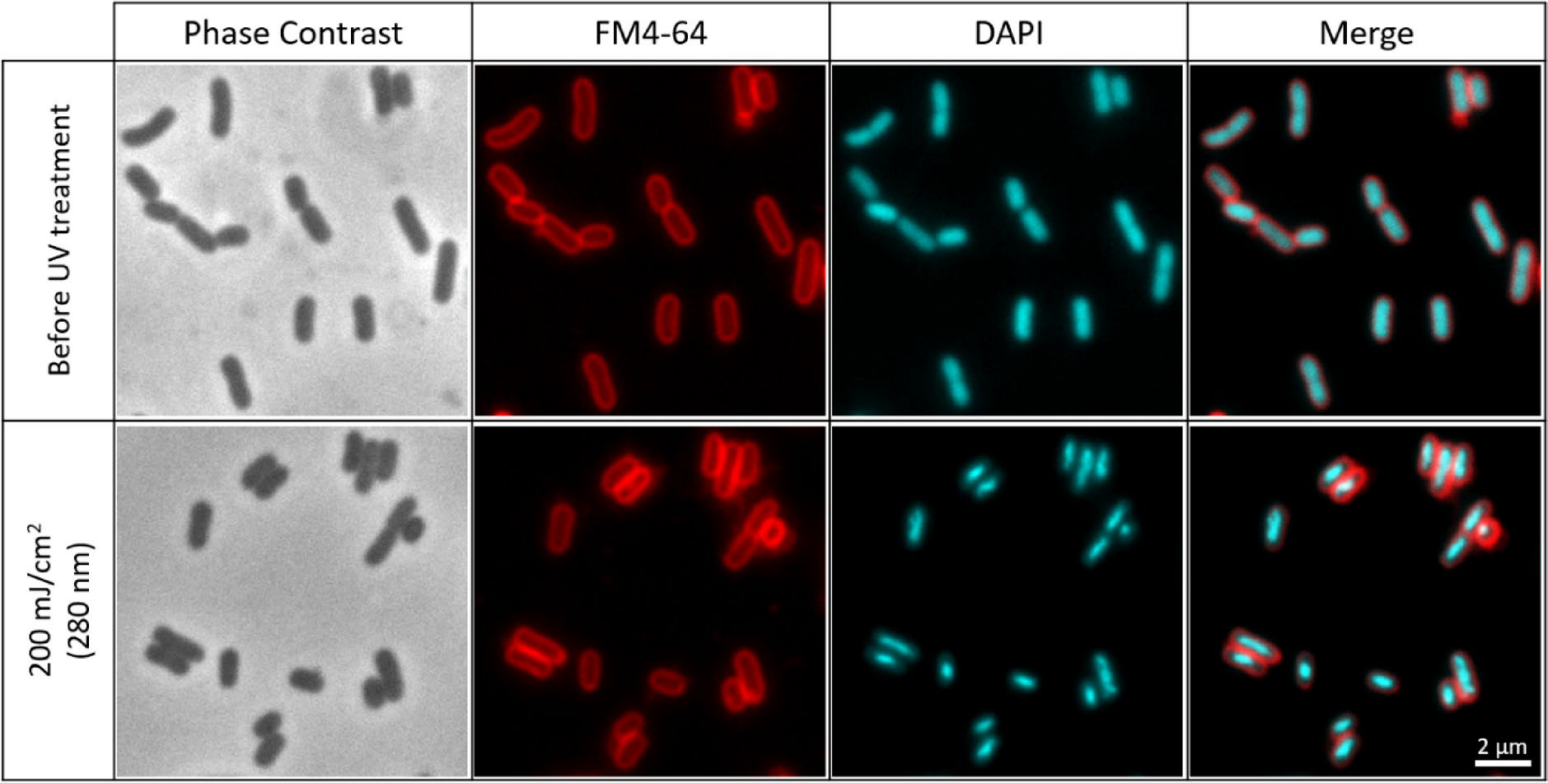
Example of exponential-phase *E. coli* nucleoid conformational change before and after exposure to 280 nm UV-C LEDs at a UV fluence of 200 mJ/cm². From the left side, the first column presents phase contrast images (grayscale), the second shows membrane staining with FM4-64 (red), the third displays DNA stained with DAPI (cyan), and the fourth shows the merged images of FM4-64 and DAPI. Noticeable changes in DNA staining by the fluorescent dye occur, transitioning from a homogeneous dispersion throughout the cell to a more concentrated and irregularly shaped nucleoid.

**Fig. 8:**
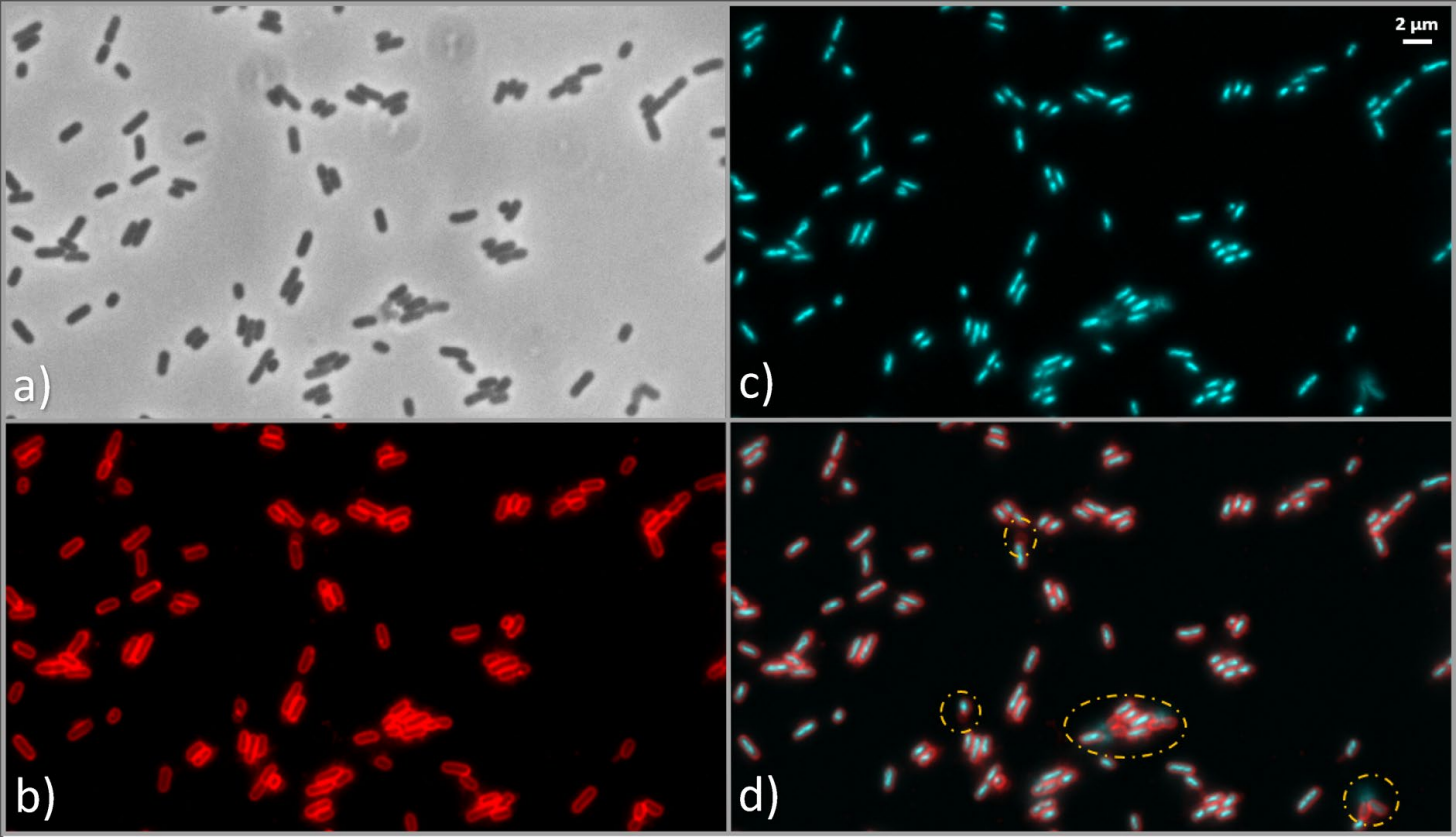
Example of exponential-phase *E. coli* cells retaining membrane staining but lacking DAPI signal after UV-C LED exposure to 280 nm UV-C LEDs at a fluence of 200 mJ/cm². Image (**a**) shows the phase contrast, (**b**) displays membrane staining with FM4-64 (red), (**c**) shows DNA staining with DAPI (cyan) and (**d**) the merged images of FM4-64 and DAPI channels. Yellow circles highlight cells with FM4-64 signal in the absence of corresponding DAPI fluorescence, illustrating cases in which membrane staining is retained while nuclear material is no longer detected by DAPI, consistent with severe perturbation of DNA structure or accessibility.

Although the inactivation experiments were primarily conducted in the stationary growth phase at a fixed UV fluence of 14 mJ/cm², additional assays extended the UV fluence up to 200 mJ/cm² to assess whether the prevalence of DNA conformational changes increased with higher UV exposure. Under both stationary and exponential growth phases of *E. coli*, DNA conformational changes were consistently observed, with more pronounced effects at higher fluences than those detected at 14 mJ/cm². Representative images of cells in the stationary and exponential phases are shown in Figs. 6 and 7, respectively. Additionally, the same phenomenon in which FM4-64 signal is retained and the DAPI is absent, can also be seen after a UV fluence of 200 mJ/cm² in Fig. 8. Moreover, larger fields of *E. coli* cells before and after exposure to 265 nm UV-C LEDs at fluences of 14 mJ/cm², 40 mJ/cm², 100 mJ/cm², and 200 mJ/cm², are shown in Figure S13 (in supplementary information section).

**Fig. 9:**
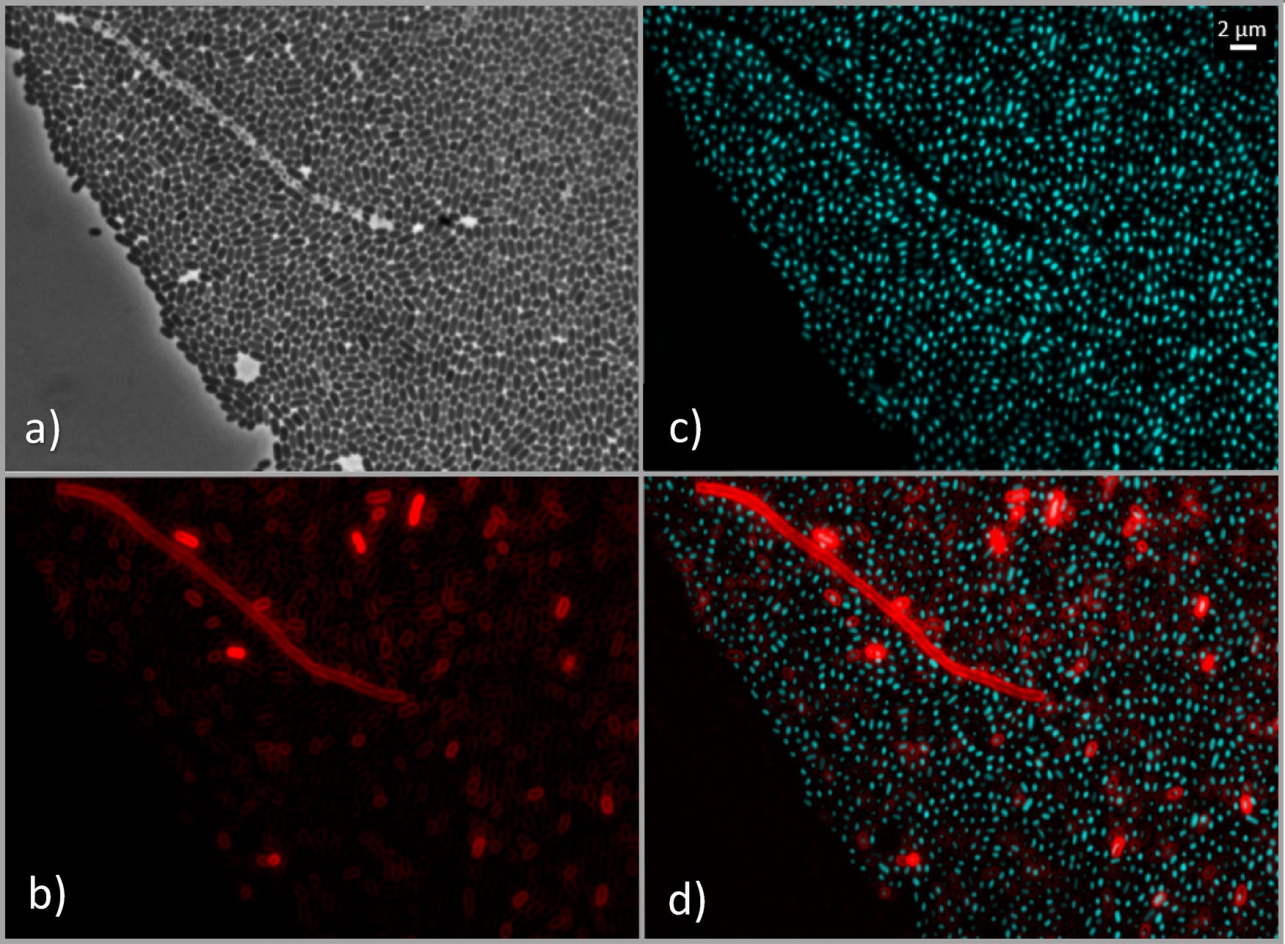
Example of stationary-phase *E. coli* filamentation stress response following exposure to 280 nm UV-C LEDs at a fluence of 14 mJ/cm² and 18-hour incubation period in absence of light. Image (**a**) shows the phase contrast, (**b**) displays membrane staining with FM4-64 (red), (**c**) shows DNA staining with DAPI (cyan) and (**d**) the merged images of FM4-64 and DAPI channels.

In addition, morphological alterations consistent with filamentation were also detected in *E. coli* following UV exposure. Figure 9 reveals an elongated cell shape, considerably longer than those observed in the untreated control samples. This phenomenon, known as filamentation, which results from cell division inhibition, is a well-documented stress response in *E. coli* and other bacteria, often triggered by DNA damage^95,96^.

These observations are in line with other studies showing that UV and other genotoxic or oxidative stresses can trigger nucleoid condensation, filamentation and large-scale rearrangements of chromosome organization in bacteria, often mediated by nucleoid-associated proteins such as HU or Dps and by SOS-driven filamentation programs^93,97,98^.

#### Quantification of DNA spatial reorganization using fluorescence intensity distribution skewness

To quantify alterations in DNA organization in *E. coli* following UV exposure, fluorescence microscopy images of DAPI-stained cells were analysed using the open-source image analysis platform Fiji. The parameter “skewness” was used to characterize the asymmetry of fluorescence intensity distributions within individual cells. Skewness provides a measure of deviation from a symmetrical (normally distributed) intensity profile. Cellpose, a deep learning-based segmentation method^99^, was used for mapping the regions of interest.

**Fig. 10:**
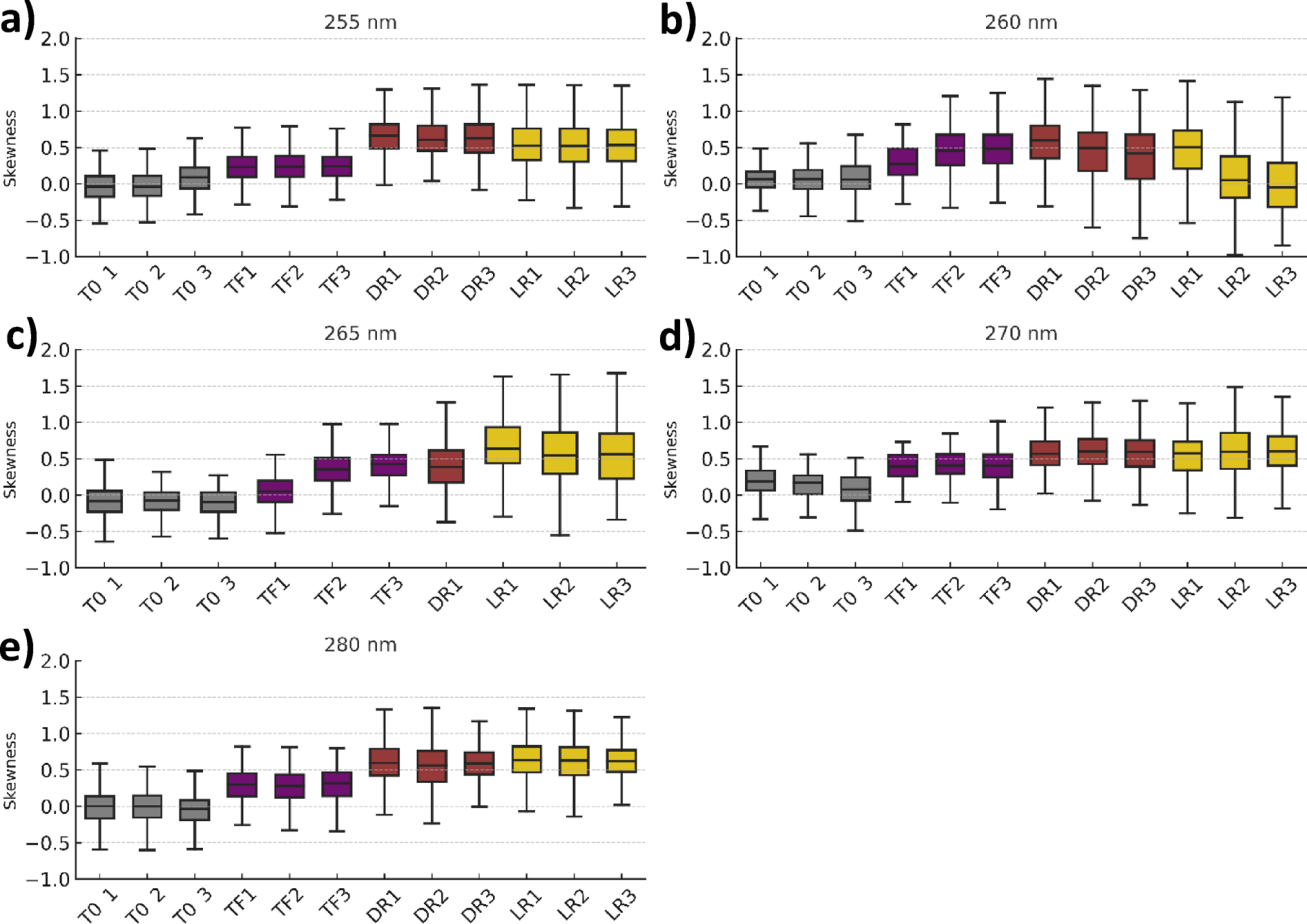
Comparison of skewness values derived from three microscopy images of *E. coli* per condition—including initial concentration (T0), post-exposure to 14 mJ/cm² (TF), dark reactivation (DR) and light reactivation (LR) —following treatment with the LEDs emitting light at 255 nm (**a**), 260 nm (**b**), 265 nm (**c**), 270 nm (**d**) and 280 nm (**e**). Upon exposure to LEDs, the skewness value generally increases across all wavelengths.

Figure 10 presents a comparison of skewness values calculated from three microscopy images per condition, including the initial bacterial suspension, post-exposure to 14 mJ/cm², dark reactivation, and light reactivation. These images were taken from distinct fields of view on the same microscopy sample and do not represent biological replicates from independent experiments.

The number of cells analyzed per image varied depending on cell density, ranging from 200 segmented cells to 1695 segmented cells. When combining all conditions and wavelengths, an average of approximately 1000 cells were analyzed per condition per wavelength. In control cells, DAPI fluorescence was typically homogeneous and uniform, resulting in histograms with low skewness values. In contrast, UV-exposed cells exhibited a more heterogeneous distribution of DNA-associated fluorescence, leading to broader, asymmetrical intensity profiles and increased skewness values.

In general, an increase in skewness values was observed after UV exposure across all UV-C LED wavelengths tested, in comparison with pre-exposure conditions. This trend suggests alterations in fluorescence signal distribution, potentially reflecting changes in DNA organization or intracellular content following UV-induced damage. Regarding the 265 nm wavelength, only one skewness value is shown for the dark reactivation condition due to low resolution in the remaining images. The consistency of the data supports the use of skewness as a sensitive and quantifiable parameter for detecting DNA conformational changes following UV-induced stress.

In UV and UV LED disinfection research, fluorescence microscopy has typically been used for qualitative assessments of membrane integrity or for coarse DAPI-based visualization of DNA damage, rather than for quantitative analysis of how nucleoid architecture changes in individual cells. In contrast, our work introduces a quantitative, single-cell measure of UV-induced nucleoid reorganization, by combining Cellpose-based segmentation and skewness analysis of the DAPI fluorescence intensity distribution. To the best of our knowledge, no previous UV LED study has quantified nucleoid conformational changes by fluorescence microscopy across different wavelengths. Further research is required to clarify the link between the staining pattern and particular types of UV-induced DNA damage. These single-cell measurements are in line with recent UV LED disinfection reviews, which emphasize the need for more detailed information on how UV exposure alters bacterial structures in addition to bulk log-reduction data^9,17^.

#### Dimer formation

**Fig. 11:**
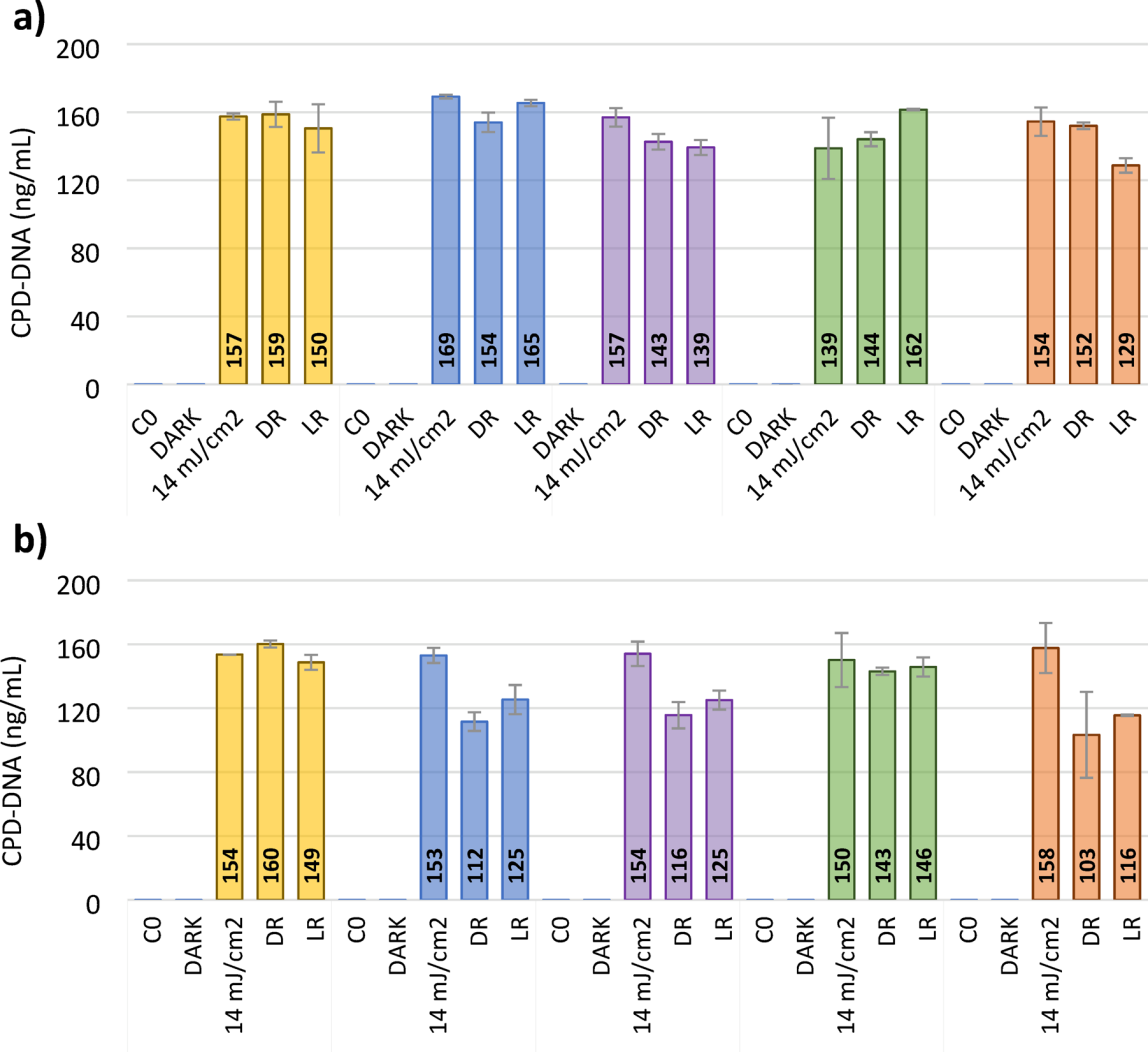
Concentration of cyclobutane pyrimidine dimers (CPDs) formed in the DNA of *E. coli* (**a**) and *E. faecium* (**b**) after exposure to a UV fluence of 14 mJ/cm² from UV LEDs that emit light at different wavelengths: 255 nm, 260 nm, 265 nm, 270 nm, and 280 nm. CPDs were not detected in the dark controls. Error bars represent duplicate results.

Inactivation by UV-C radiation occurs primarily through the induction of DNA damage in microorganisms, with cyclobutane pyrimidine dimers being among the most predominant lesions formed^24,75^. To quantify UV-induced DNA damage, the concentration of CPDs was measured following the application of a UV fluence of 14 mJ/cm² using LEDs emitting at different wavelengths. Figure 11 displays the concentrations of cyclobutane pyrimidine dimers detected in the DNA of both environmental strains of *E. coli* and *E. faecium* following exposure to UV-C LEDs at different wavelengths, as well as after incubation under light (photoreactivation) and dark (dark repair) conditions.

The results obtained show that similar CPD levels were formed in the target bacteria after exposure to the different wavelengths tested.

Subsequent assessments of CPD concentrations after 18 h of incubation under dark or light conditions showed that both microorganisms typically maintained the CPD levels formed, although in the reactivation experiments of *E. faecium* after exposure to LEDs that emit at 260 nm, 265 nm and 280 nm a decrease in the pyrimidine dimers formed was observed.

The apparent discrepancy between the greater ability of *E. faecium* to repair cyclobutane pyrimidine dimers and the absence of reactivation in both species following inactivation assays (Figure 2) indicates that the removal of DNA lesions alone does not necessarily ensure cellular recovery. Although *E. faecium* demonstrates more efficient CPD repair, this advantage does not translate into measurable reactivation under the tested conditions.

Similar findings have been reported in previous studies. For example, in a qPCR-based analysis, Süß et al.^24^ observed that *E. faecium* did not display detectable DNA repair following exposure to low-pressure mercury lamps, despite initial CPD formation. Even in cases where CPD levels declined over time, the colony-forming ability remained suppressed, suggesting that DNA damage repair alone is insufficient for complete cellular recovery. McKinney and Pruden^25^ investigated reactivation in multiple strains of enterococci, including environmental and clinical isolates of *E. faecalis* and *E. faecium*, following exposure to UV disinfection. They found high variability in repair capacity between strains and noted that photoreactivation did not necessarily lead to restored culturability, particularly in *E. faecium*, highlighting strain-dependent differences and the potential disconnect between DNA lesion repair and viability. Similarly, Chen et al.^26^ studied *E. coli*, *L. monocytogenes*, and *E. faecium* under combined UV and chlorine disinfection. While DNA damage from UV treatment was partially repaired in some cases, especially in *E. faecium*, recovery of viability was not consistently observed.

These results combined indicate that factors beyond DNA repair, such as damage to other cellular structures or limitations in the recovery machinery, may constrain the reactivation potential following UV-C LED exposure, highlighting the complex nature of microbial stress responses and the treatment efficiency.

## Conclusions

Similar inactivation kinetics were observed between environmental and culture collection strains of *E. coli* and *E. faecium* across all the tested UV-C LED wavelengths, with the highest inactivation efficiency consistently achieved at 265 nm. This consistency across strains highlights the robustness of UV-C LED disinfection, particularly at this wavelength.

Despite the potential for environmental isolates to develop increased tolerance to UV radiation due to prolonged exposure to sunlight, the environmental isolates of *E. coli* and *E. faecium* tested in this study demonstrated similar UV susceptibility in comparison to culture collection strains. These results highlight the broad effectiveness of UV-C LED treatment against both laboratory and environmentally derived bacteria, reinforcing its potential as a reliable disinfection strategy in real-world water treatment applications.

Neither *E. coli* nor *E. faecium* demonstrated substantial reactivation after LED exposure to 14 mJ/cm² under either light or dark conditions. This suggests that the treatment induced extensive and irreversible damage, potentially exceeding cyclobutane pyrimidine dimer formation and involving membrane disruption or oxidative modifications. Although *E. faecium* displayed a greater capacity for CPD repair than *E. coli*, neither microorganism regained detectable cultivability, indicating that partial DNA repair alone was insufficient to ensure cellular recovery. These results suggest that the applied UV-C dose was sufficient to overcome cellular repair mechanisms, resulting in robust inactivation of both organisms.

Fluorescence microscopy analysis revealed consistent alterations in DNA conformation upon exposure to UV-C LEDs, with a stronger effect at higher UV fluences. Most cells appeared to retain membrane integrity; however, a subset showed loss of DAPI fluorescence together with evidence of membrane damage, suggesting a strong disturbance of normal DNA staining in damaged cells. At higher fluences, DAPI staining became increasingly heterogeneous, consistent with nucleoid remodelling. Complementary staining with SYTO9 and PI confirmed increased membrane susceptibility upon higher UV fluences. In addition, filamentation was observed in *E. coli* following UV exposure. This phenomenon is typically associated with suppression of cell division in response to DNA damage, supporting the induction of stress-related pathways upon UV-C irradiation.

Overall, the outcomes of this investigation confirm the promising disinfection effectiveness of UV-C LEDs for inactivating key water quality indicator bacteria. Despite differences in cell morphology and environmental origin, both *E. coli* and *E. faecium* exhibited similar responses in terms of inactivation kinetics and post-irradiation repair. In addition, the skewness-based single-cell analysis of DAPI-stained nucleoids proposed in this work, provides a novel quantitative microscopy metric of UV-induced DNA reorganization that complements conventional viability and CPD measurements and can be applied in future UV disinfection studies.

## Supplementary Information

Below is the link to the electronic supplementary material.

Supplementary Material 1

## Data Availability

Data is provided within the manuscript or supplementary information files.
