# Supplementary material for: UV-C LED wavelength effects on inactivation kinetics, DNA damage and membrane integrity in drinking water indicator bacteria
Source: Sci Rep. 2026 Apr 3;16:15919. doi: 10.1038/s41598-026-44556-8 (PMC13195164; doi:10.1038/s41598-026-44556-8)
Supplement: Supplementary file 1 — Supplementary Material 1 [file 41598_2026_44556_MOESM1_ESM.docx]

**Supplementary Information Section**

**UV-C LED wavelength effects on inactivation kinetics, DNA damage and membrane integrity in drinking water indicator bacteria**

João Sério^1,2^, Carolina Santos^1,2^, Maria Eduarda Martins^1^, Ana Paula Marques^1^, Carolina Feliciano^2^, Mónica Serrano^2^, Adriano O. Henriques^2^, Maria Teresa Barreto Crespo^1,2^, Vanessa Jorge Pereira^1,2*^

^1^iBET, Instituto de Biologia Experimental e Tecnológica, Apartado 12, 2781-901 Oeiras, Portugal

^2^Instituto de Tecnologia Química e Biológica António Xavier, Universidade Nova de Lisboa, Av. da República, 2780-157 Oeiras, Portugal

*email corresponding author: [vanessap@ibet.pt](mailto:vanessap@ibet.pt)

S1 - Characterization of UV LEDs and determination of UV fluence

To enable robust comparison among experiments, the UV fluence (mJ/cm²) was determined as the product of the corrected incident irradiance of the UV LEDs and the exposure time in seconds. Before each inactivation experiment, the light intensity of each LED set was measured with the ILT950-UV radiometer at 4 cm from the light source (the same distance used during inactivation; see **Figures S2 and S3** in supplementary information section). Correction factors were applied to account for reflection factor (0.90), Petri factor (0.98), divergence factor (0.64), and a water factor based on sample absorption as described by Bolton and Linden [1]. A metallic reactor support was custom built (shown in **Figure S3** in supplementary information section) to ensure that the LED reactors were always placed in the same position compared to the radiometer to guarantee a higher consistency in the light intensity measurements taken in different days.

The LEDs spectral information, including peak wavelength, full width at half maximum and spectral characterization are summarized in **Table S1** (supplementary information section). All LEDs exhibited near-monochromatic output with peak emissions close to their nominal wavelengths and relatively narrow full width at half maximum, with limited emission outside the main UV-C band. The 265 and 270 nm LEDs displayed partial spectral overlap, but their peak positions and spectral shapes remained distinct and correspond to separate commercial LEDs.

In parallel work, we have analysed the wavelength-specific transcriptomic response of the same environmental *E. coli* isolate exposed to UV-C LEDs at 255, 260, 265, 270 and 280 nm using RNA-Seq [2]. Although all wavelengths triggered a common UV-C core response, despite their similar spectral output the 265 and 270 nm LEDs produced clearly distinct transcriptional fingerprints, with 265 nm showing a more DNA-centred stress pattern and 270 nm a stronger envelope/proteostasis signature. These differences indicate that even small shifts in peak emission and spectral width, such as those between the 265 and 270 nm LEDs, can lead to distinct cellular responses, supporting their analysis.

The observed light intensity variation across different wavelengths highlights the critical need to report the inactivation results as a function of UV fluence, thereby enabling accurate comparisons among LED systems emitting at distinct wavelengths and allowing a clear understanding of the wavelength-dependent effects on inactivation efficacy. This practice guarantees a reliable comparison of results between different experiments and with inactivation data reported in the literature.

According to Martín-Sómer *et al*. [3], LEDs emitting at 280 nm exhibit higher intensity than those at 260 nm and 270 nm due to differences in quantum efficiency and electricity-to-photon conversion efficiency. Although the 260 nm wavelength overlaps with the maximum UV absorbance of nucleic acids [4,5], LEDs at this wavelength typically have low wall-plug efficiency, resulting in greater energy loss as heat. In contrast, 280 nm UV LEDs, while less effective per photon in targeting DNA, offer superior electrical-to-light conversion efficiency and can achieve higher overall intensities, making them more suitable to achieve a high inactivation efficiency with reduced energy consumption, as discussed by Li *et al*. [6].

Furthermore, other studies indicate that UV-C LED intensity may decline over time due to temperature effects and operational conditions, resulting in the need for progressively longer exposure times to achieve the same bacterial inactivation levels [7-9], highlighting the importance of measuring light intensity prior to each set of experiments and converting it to UV fluence.

S2 - Isolation of *E. coli* and *E. faecium* from Surface Water Samples

Strains of *E. coli* and *E. faecium*, widely used as water quality indicators of fecal contamination [10,11], were isolated from surface water samples using membrane filtration followed by cultivation on selective chromogenic media.

Water samples were processed using a sterile filtration system (Nalgene®, Rochester, NY, USA) connected to a vacuum pump (Cole-Parmer, Vernon Hills, IL, USA). The filtration was performed using 47 mm diameter membranes with an average pore size of 0.45 µm (MF-Millipore™ gridded). Environmental isolates of *E. coli* and *E. faecium* were obtained using selective chromogenic media. *E. coli* was isolated on RAPID’E. coli 2 Medium (Bio-Rad), which differentiates colonies based on β-D-glucuronidase and β-D-galactosidase activities, enabling the identification of *E. coli* (GAL⁺/GLUC⁺) as violet to pink colonies, while other coliforms appear blue. The medium’s high specificity is enhanced by selective inhibition of background flora, particularly at 44 °C. *E. faecium* was isolated using *E. faecium* ChromoSelect Agar (Sigma-Aldrich), which relies on the arabinose-fermenting capability of *E. faecium*, resulting in green colonies and a characteristic yellow halo due to enzymatic cleavage of a chromogenic substrate. The medium is supplemented with aztreonam to suppress the growth of Gram-negative bacteria, including *Pseudomonas aeruginosa* and *E. coli*, ensuring selective recovery from complex environmental samples.

After isolation, the identification of the environment isolates was confirmed through 16S rDNA gene sequencing performed by STAB Vida (Portugal). The sequence of the 16S rRNA gene of each isolate was compared with those available in the GenBank database using the Basic Local Alignment Search Tool (BLAST) program from the National Centre for Biotechnology Information (NCBI) (<http://www.ncbi.nlm.nih.gov>). The results obtained confirmed the similarity of the sequences to those of *E. coli* and *E. faecium* strains in the GenBank database.

S3 - Data Analysis

The inactivation efficiency was assessed experimentally by quantifying the reduction in viable bacterial concentrations —expressed as colony forming units per milliliter (CFU/mL)— as a function of the applied UV fluence. The constant *k* (kt or kf) represents the pseudo first order direct photolysis inactivation rate constant, which can be calculated as the slope of a linear regression of the log inactivation as a function of time or UV fluence, as shown by the equation:

$$\log\left( \frac{Ci}{Cf} \right)=kf \times UV fluence$$

where *Ci* and *Cf* represent the concentrations of viable cells prior to and following UV-C LED exposure, respectively.

Supplementary Section Figures


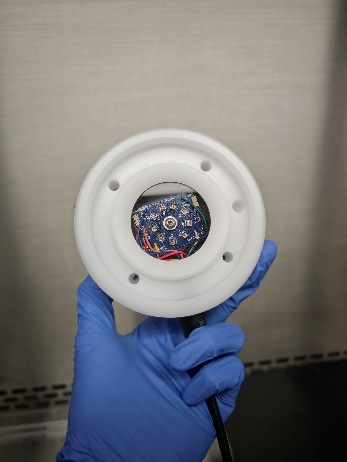

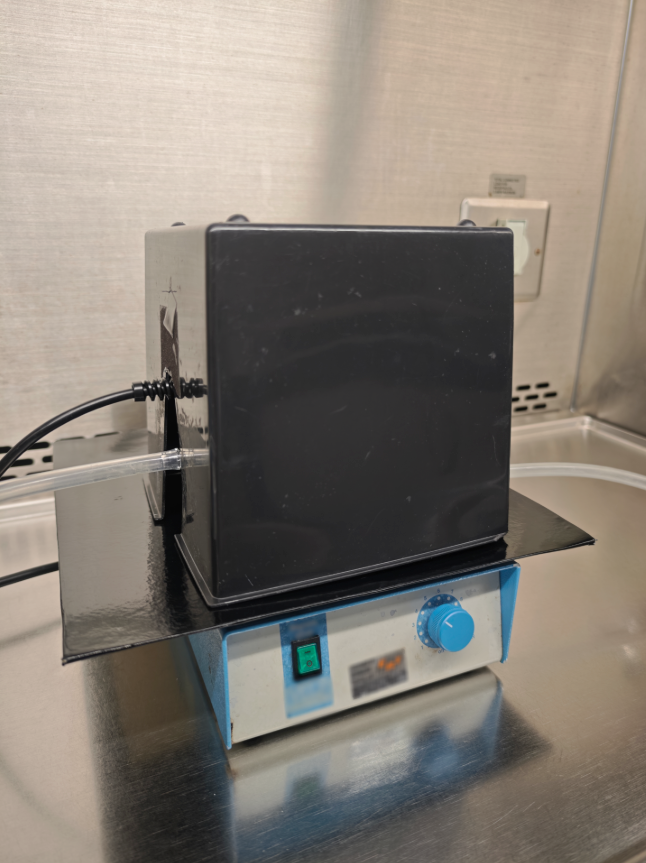

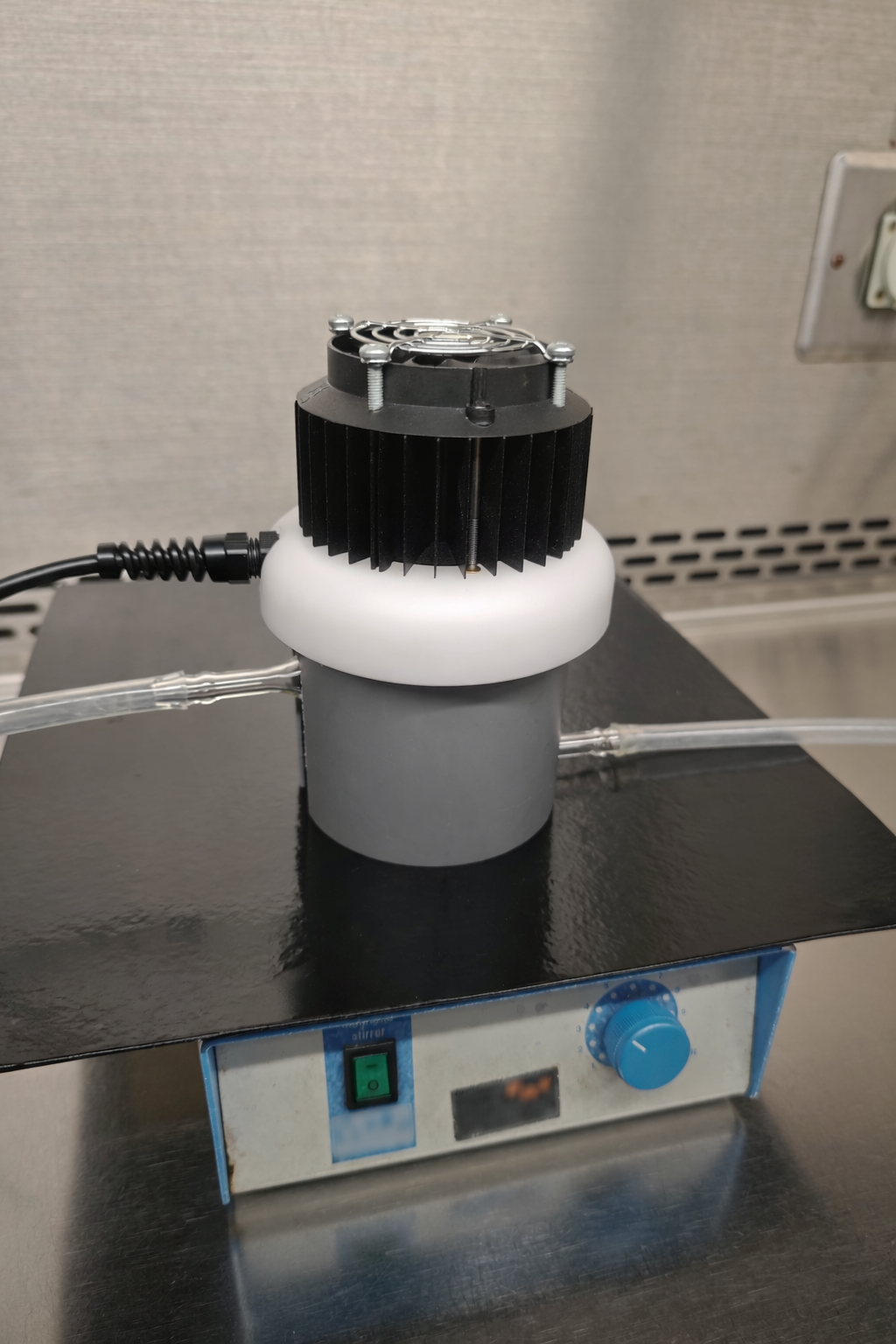

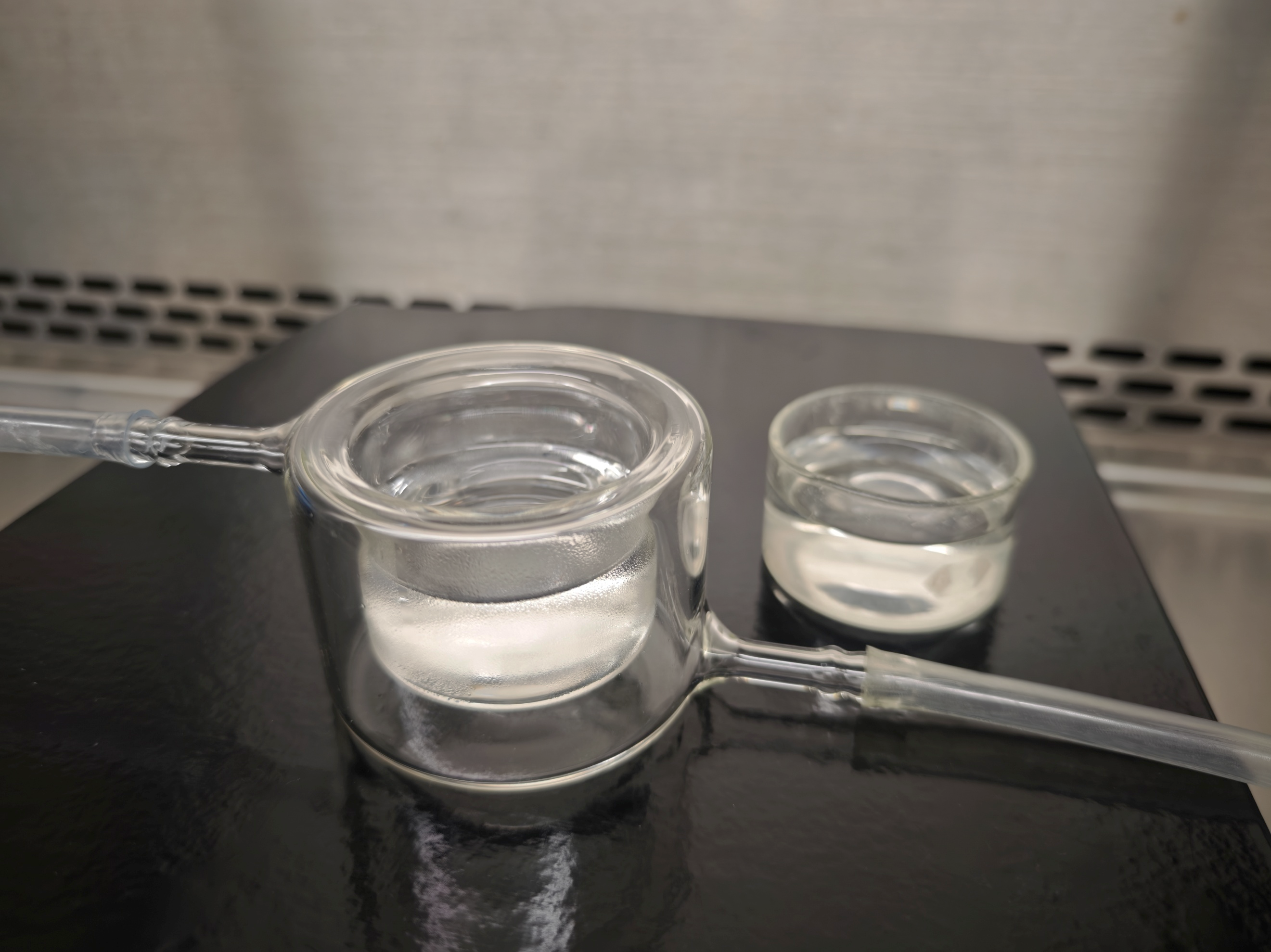


**a)**

**c)**

**b)**

**d)**

**[2]**

**[1]**

**Figure S1** - Inactivation experiments setup using UV-C light-emitting diodes. a) UV LED reactor (AquiSense Technologies, USA). b) [1] Refrigerated and continuously stirred sample container; [2] Dark control. c) Cylindrical stand for the UV-LED reactor. d) Black box to contain radiation emitted during the experiment.

**Figure S2** - Light intensity of the LEDs used in this study emitting light at different wavelengths [I_280 nm_ > I_260 nm_ ~ I_270 nm_ > I_265 nm_ > I_255nm_]


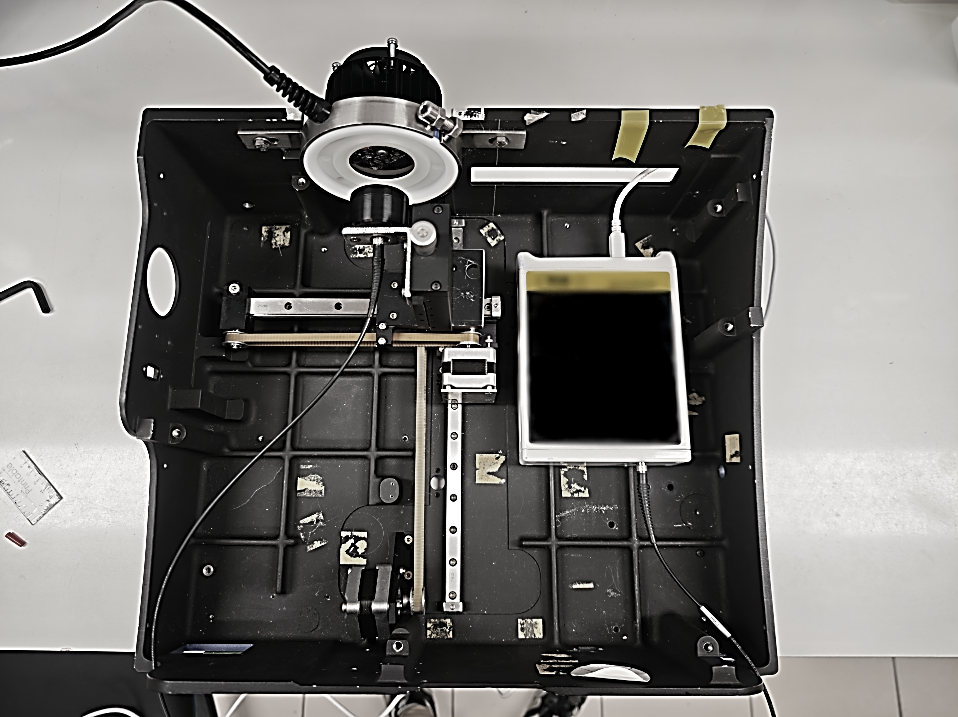


4 cm distance between LED

reactor and radiometer

A

B

B

**Figure S3** - Measurement system for LED radiation intensity. A – UV LED Reactor (AquiSense Technologies, USA). B – Radiometer (SpectriLight International Light Technologies ILT950-UV).


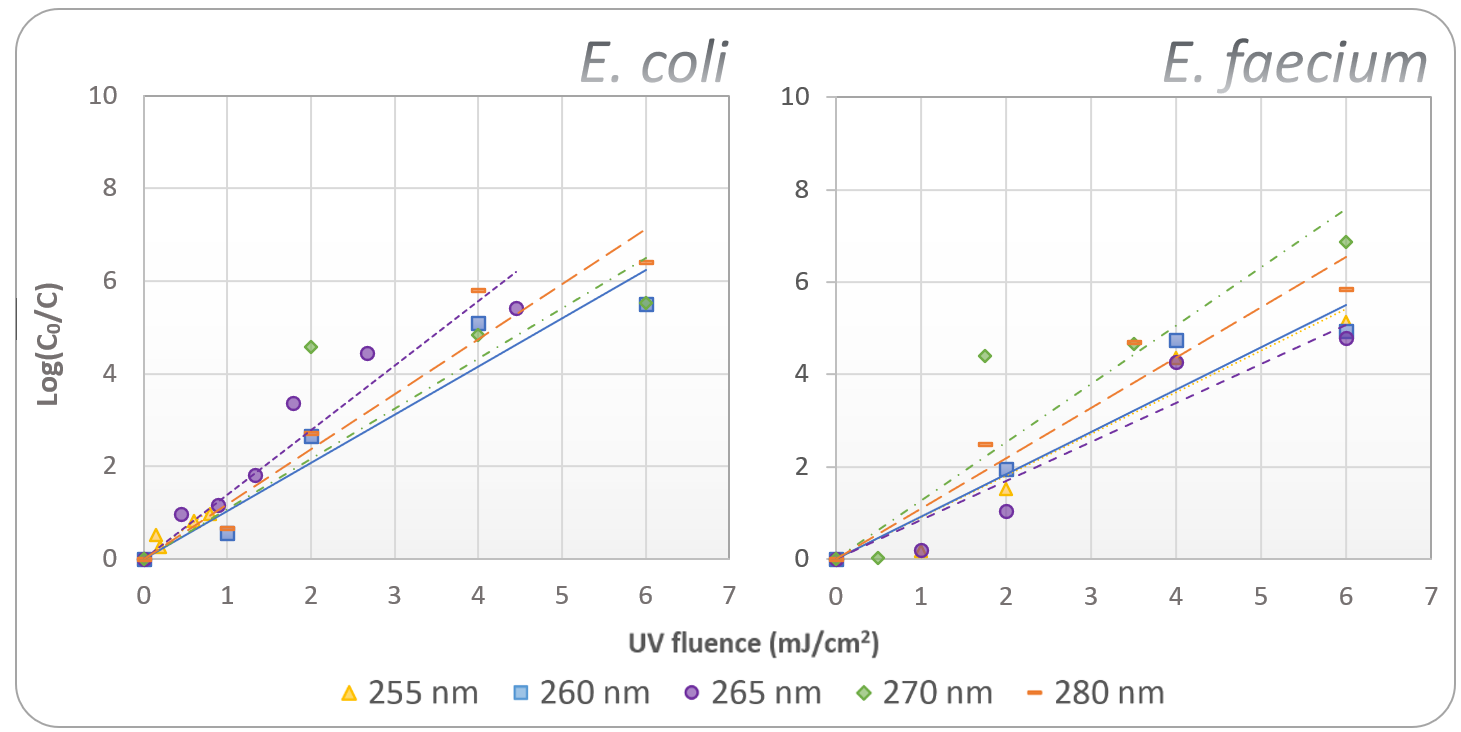


*Escherichia coli*

*Enterococcus faecium*

**Figure S4** - Inactivation of culture collection strains of *Escherichia coli and Enterococcus faecium* [log (C_0_/C)] as a function of UV fluence using LEDs that emit light at different wavelengths: 255, 260, 265, 270, and 280 nm.


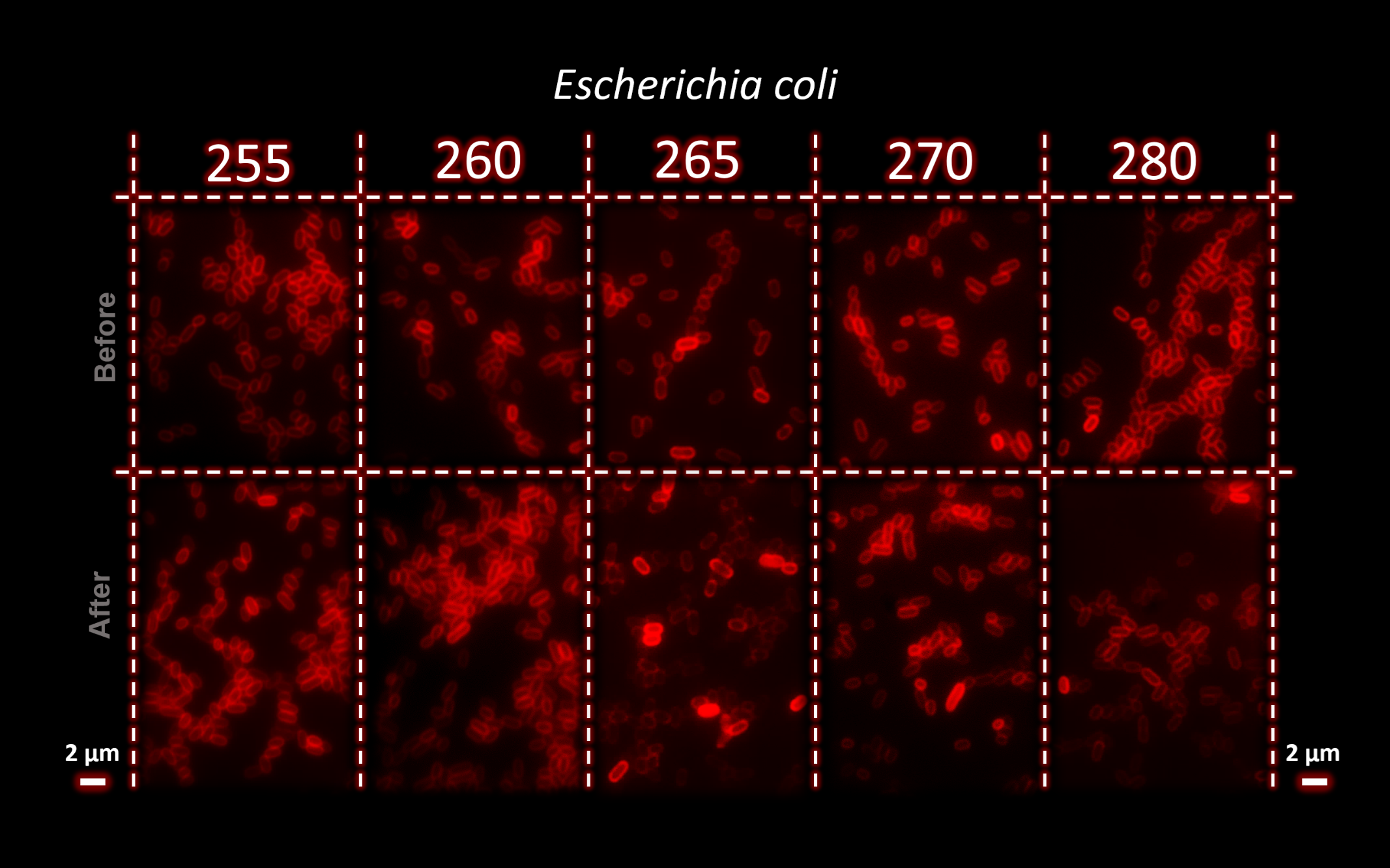


**Figure S5** – Fluorescence microscopy images of *E. coli* membranes (stained with FM4-64) before and after exposure to UV-C LEDs emitting light at 255 nm, 260 nm, 265 nm, 270 nm and 280 nm (at a UV fluence of 14 mJ/cm²). Samples were obtained before irradiation (top row) and after exposure to different wavelengths (bottom row). Each column corresponds to the indicated LED wavelength. Cells generally appear to retain membrane integrity.


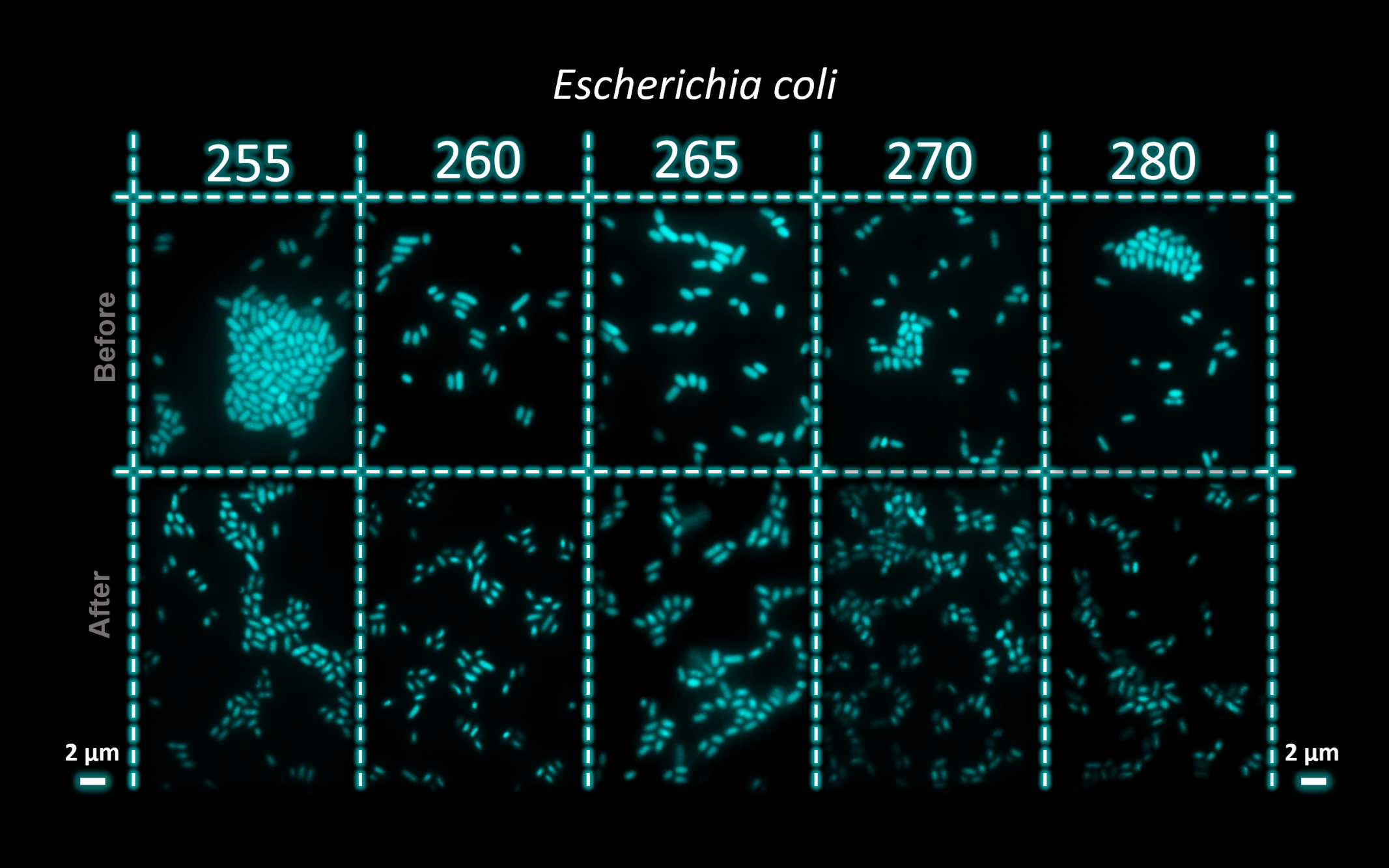


**Figure S6** - Fluorescence microscopy images of *E. coli* DNA (stained with DAPI) before and after exposure to UV-C LEDs emitting light at 255 nm, 260 nm, 265 nm, 270 nm and 280 nm (at a UV fluence of 14 mJ/cm²). Samples were obtained before irradiation (top row) and after exposure to different wavelengths (bottom row). Each column corresponds to the indicated LED wavelength. Alterations in the dispersion of DAPI can be noticed after exposure to radiation.


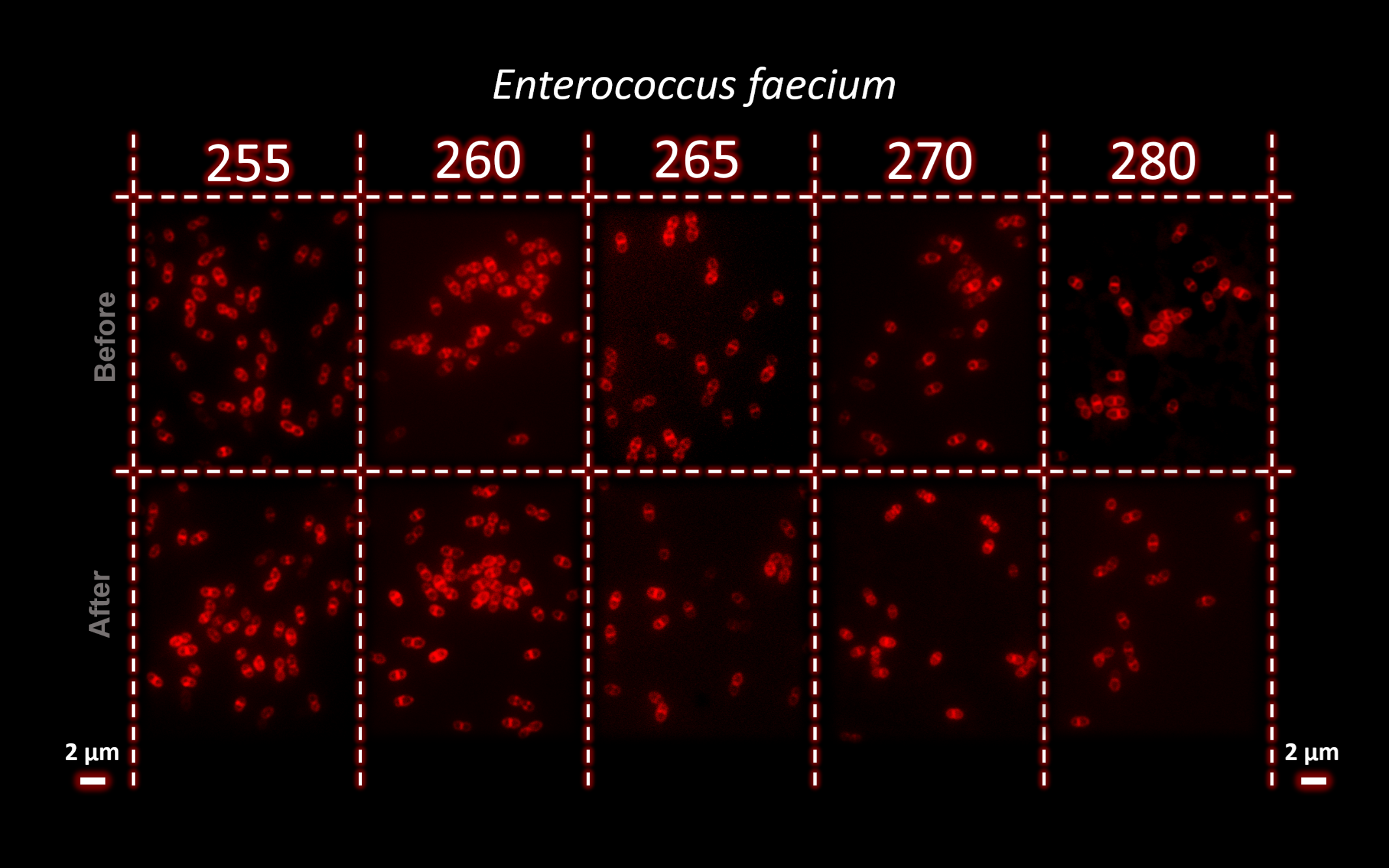


**Figure S7** - Fluorescence microscopy images of *E. faecium* membranes (stained with FM4-64) before and after exposure to UV-C LEDs emitting light at 255 nm, 260 nm, 265 nm, 270 nm and 280 nm (at a UV fluence of 14 mJ/cm²). Samples were obtained before irradiation (top row) and after exposure to different wavelengths (bottom row). Each column corresponds to the indicated LED wavelength. Cells generally appear to retain membrane integrity.


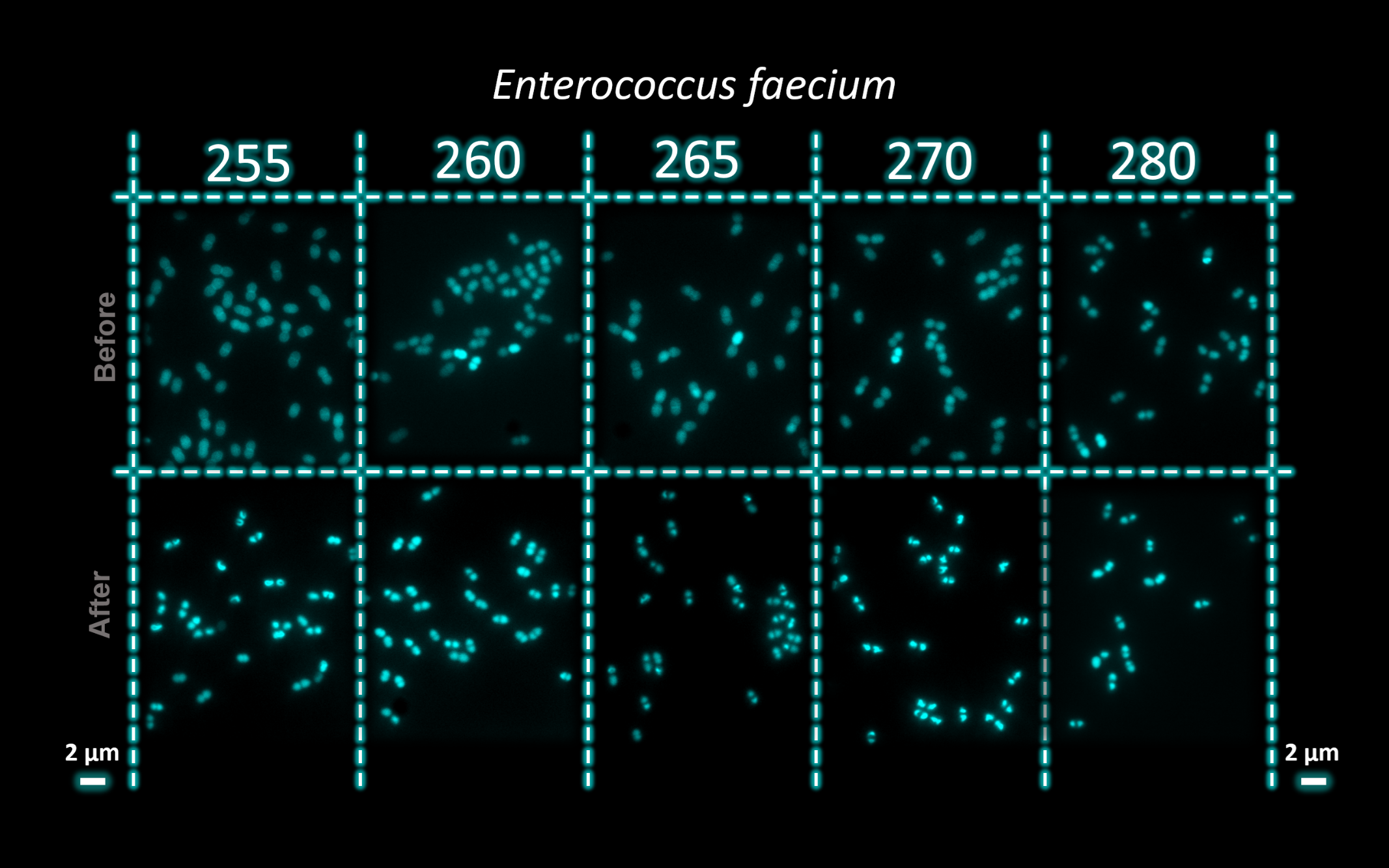


**Figure S8** - Fluorescence microscopy images of *E. faecium* DNA (stained with DAPI) before and after exposure to UV-C LEDs emitting light at 255 nm, 260 nm, 265 nm, 270 nm and 280 nm (at a UV fluence of 14 mJ/cm²). Samples were obtained before irradiation (top row) and after exposure to different wavelengths (bottom row). Each column corresponds to the indicated LED wavelength. Alterations in the dispersion of DAPI can be noticed after exposure to radiation.


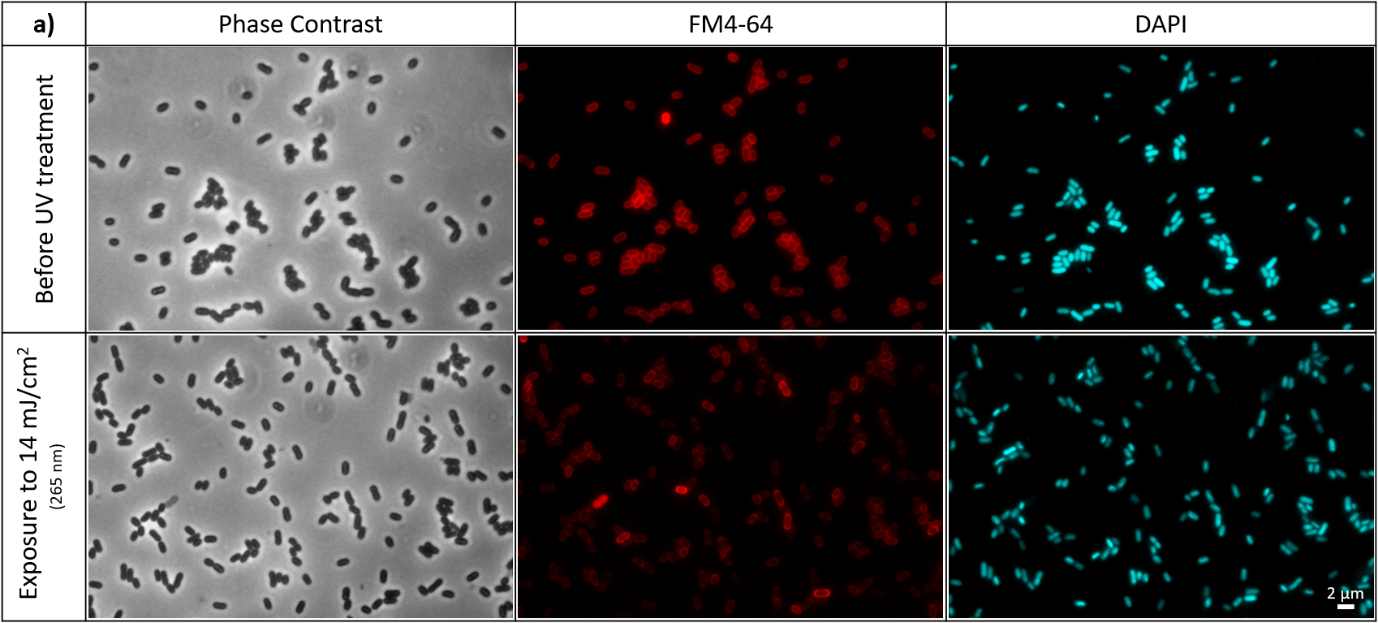

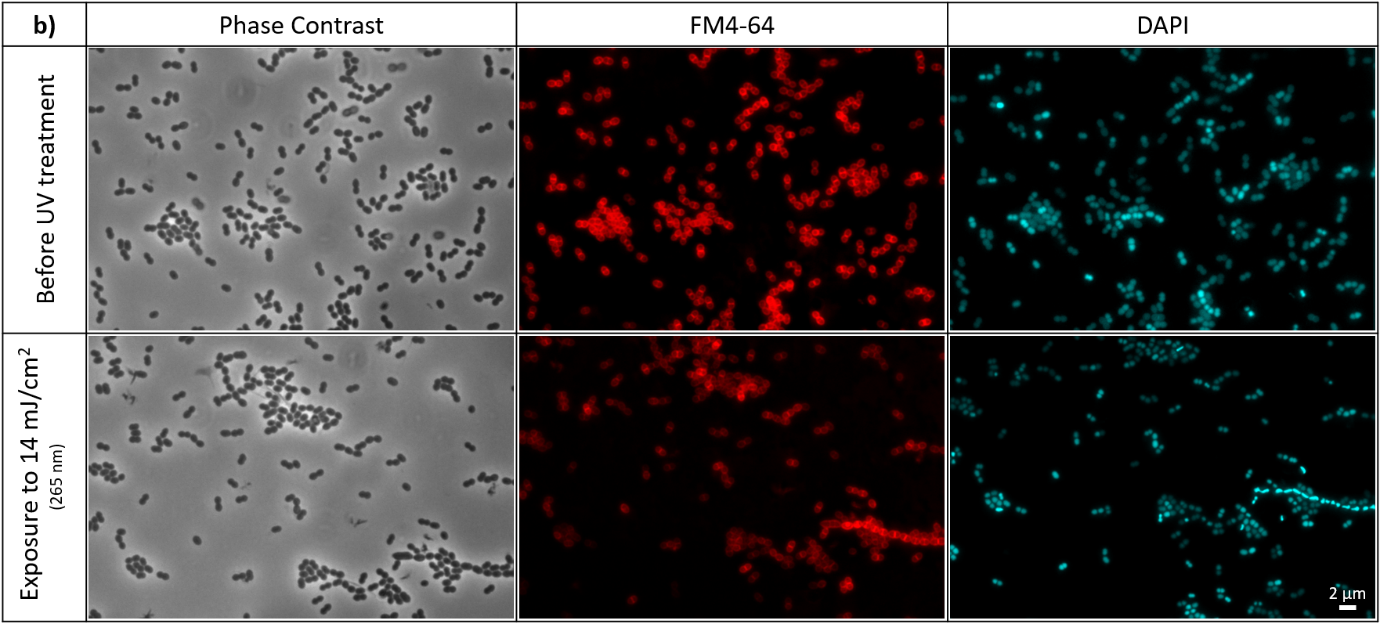


**Figure S9 -** Overview of FM4-64/DAPI staining patterns of *E. coli* (a) and *E. faecium* (b) before and after exposure to LEDs emitting at 265 nm (UV fluence of 14 mJ/cm²). The first column shows phase contrast microscopy images (grayscale), the second column displays membranes stained with FM4-64 dye (red), and the third column shows DNA stained with DAPI (blue). These images represent wider fields of view corresponding to the close-ups shown in Figure 3.


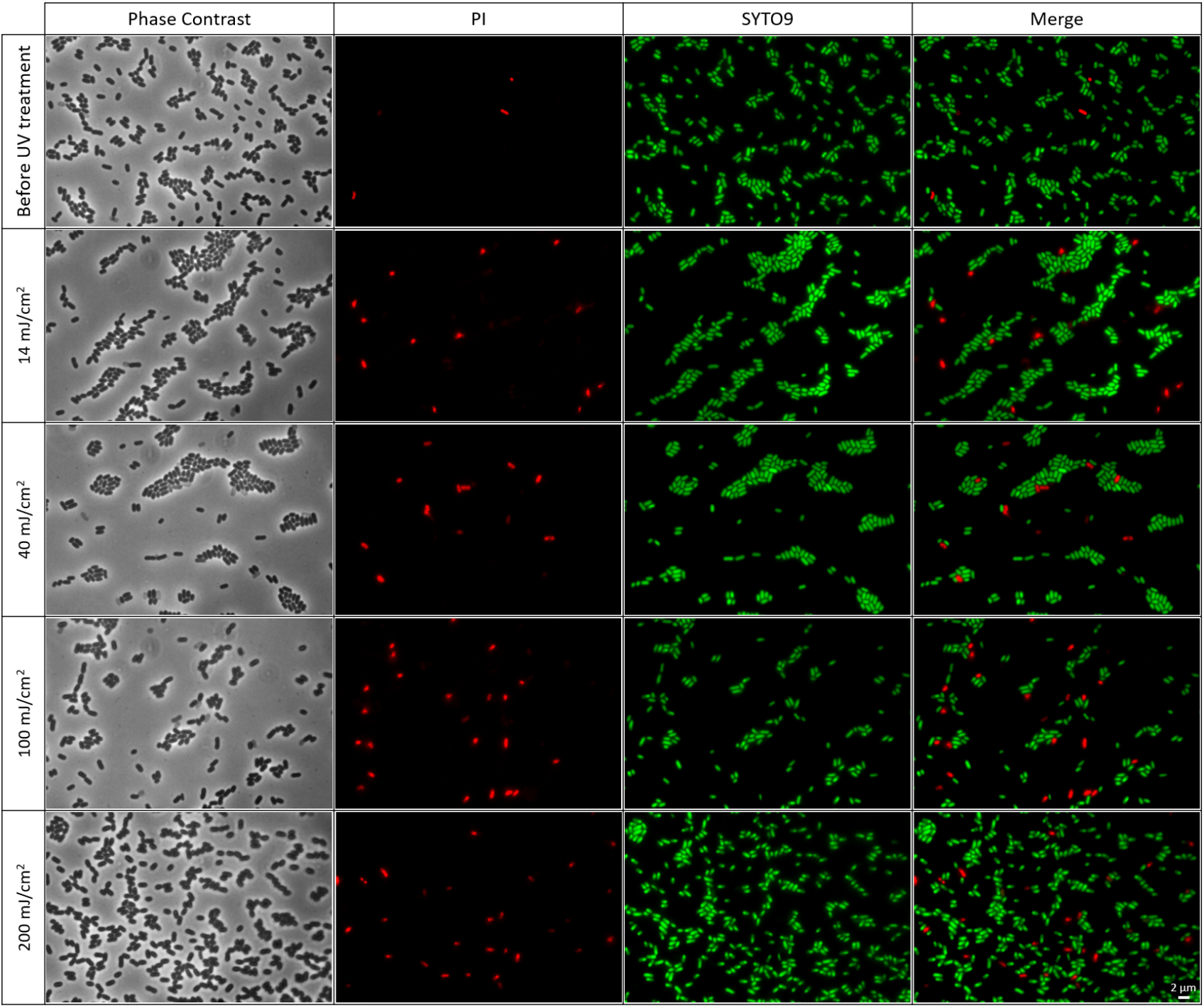


**Figure S10 -** Overview of SYTO9/propidium iodide staining of *E. coli* before and after exposure to 265 nm UV-C LEDs at UV fluences of 14 mJ/cm², 40 mJ/cm², 100 mJ/cm², and 200 mJ/cm². From the left side, the first column presents phase contrast images (grayscale), second shows non-viable cells stained with propidium iodide (red), the third shows viable cells stained with SYTO9 (green), and the fourth shows the merged images of PI and SYTO9 channels. These images represent wider fields of view corresponding to the close-ups shown in Figure 5.


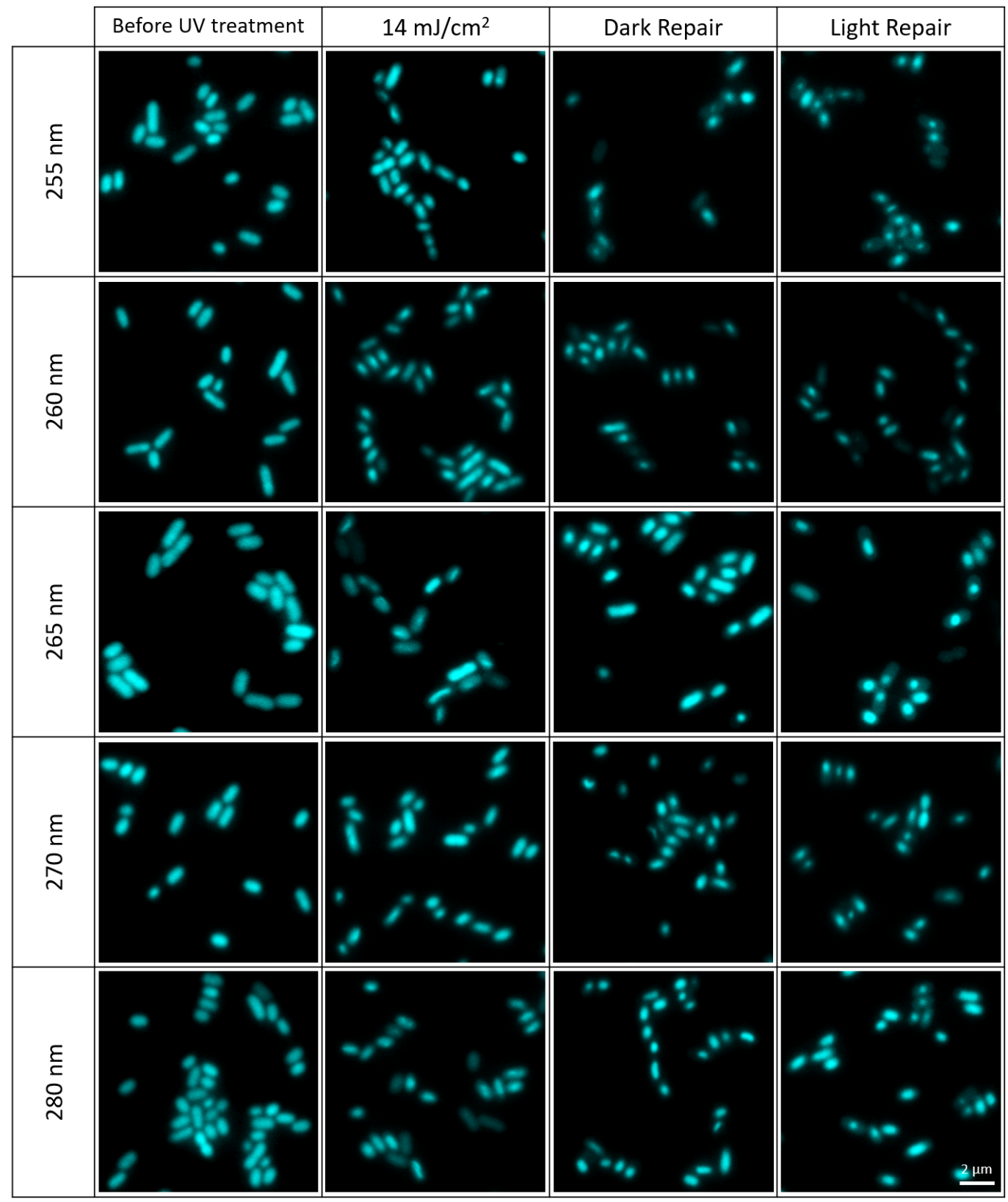


**Figure S11 –** Wavelength-dependent alterations in DAPI-stained nucleoids of *E. coli* prior to UV exposure (T0), immediately following treatment with a UV fluence of 14 mJ/cm², and after a 18-hour incubation under dark (DR) and light (LR) conditions using LEDs emitting at 255 nm, 260 nm, 265 nm, 270 nm and 280 nm. UV treatment induces alterations in DNA organization, with DAPI fluorescence shifting from a homogeneous intracellular distribution to a more condensed and irregularly shaped pattern. No reversal was observed after 18h of incubation.


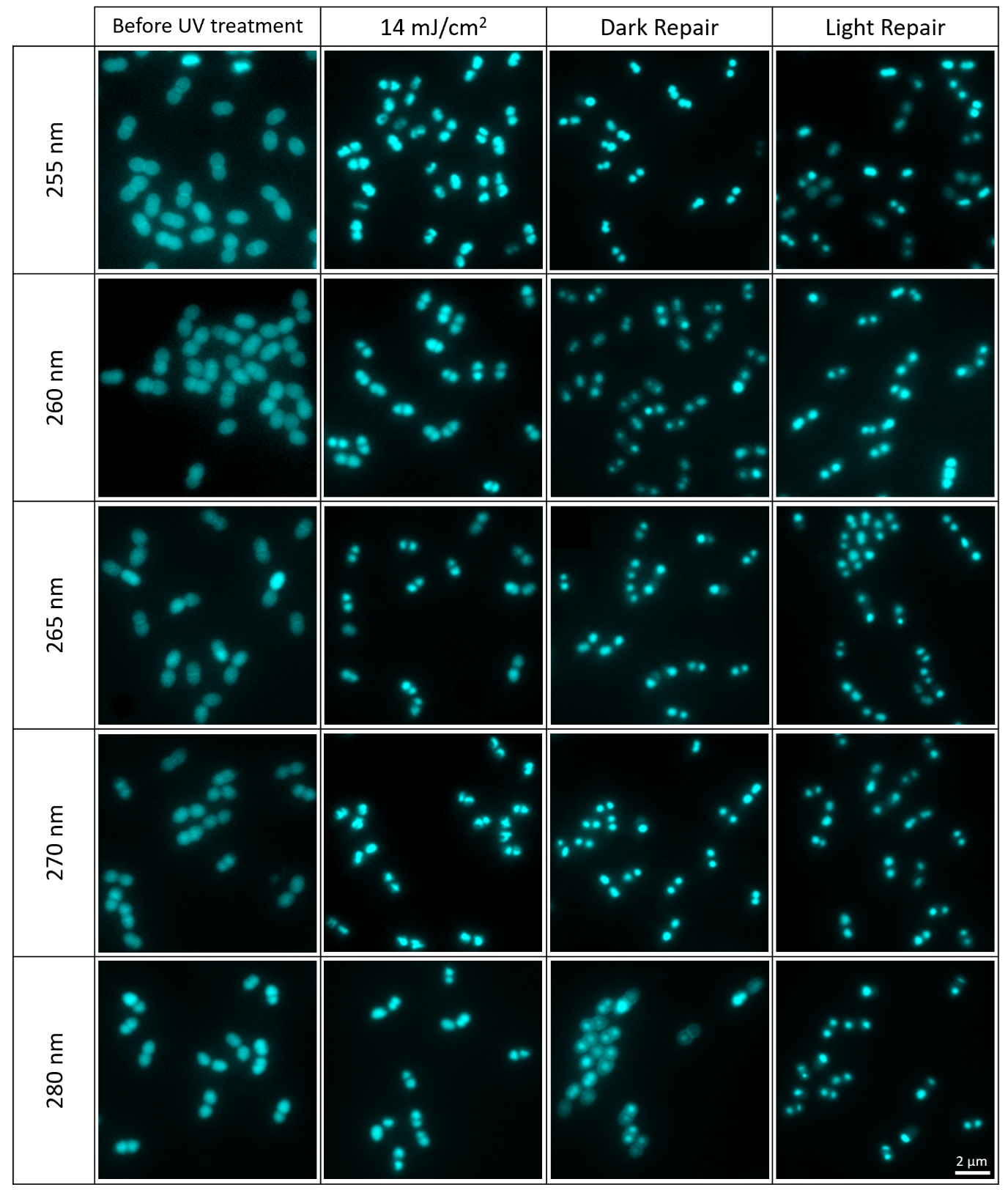


**Figure S12 –** Wavelength-dependent alterations in DAPI-stained nucleoids of *E. faecium* prior to UV exposure (T0), immediately following treatment with a UV fluence of 14 mJ/cm², and after a 18-hour incubation under dark (DR) and light (LR) conditions using LEDs emitting at 255 nm, 260 nm, 265 nm, 270 nm and 280 nm. UV treatment induces alterations in DNA organization, with DAPI fluorescence shifting from a homogeneous intracellular distribution to a more condensed and irregularly shaped pattern. No reversal was observed after 18h of incubation.


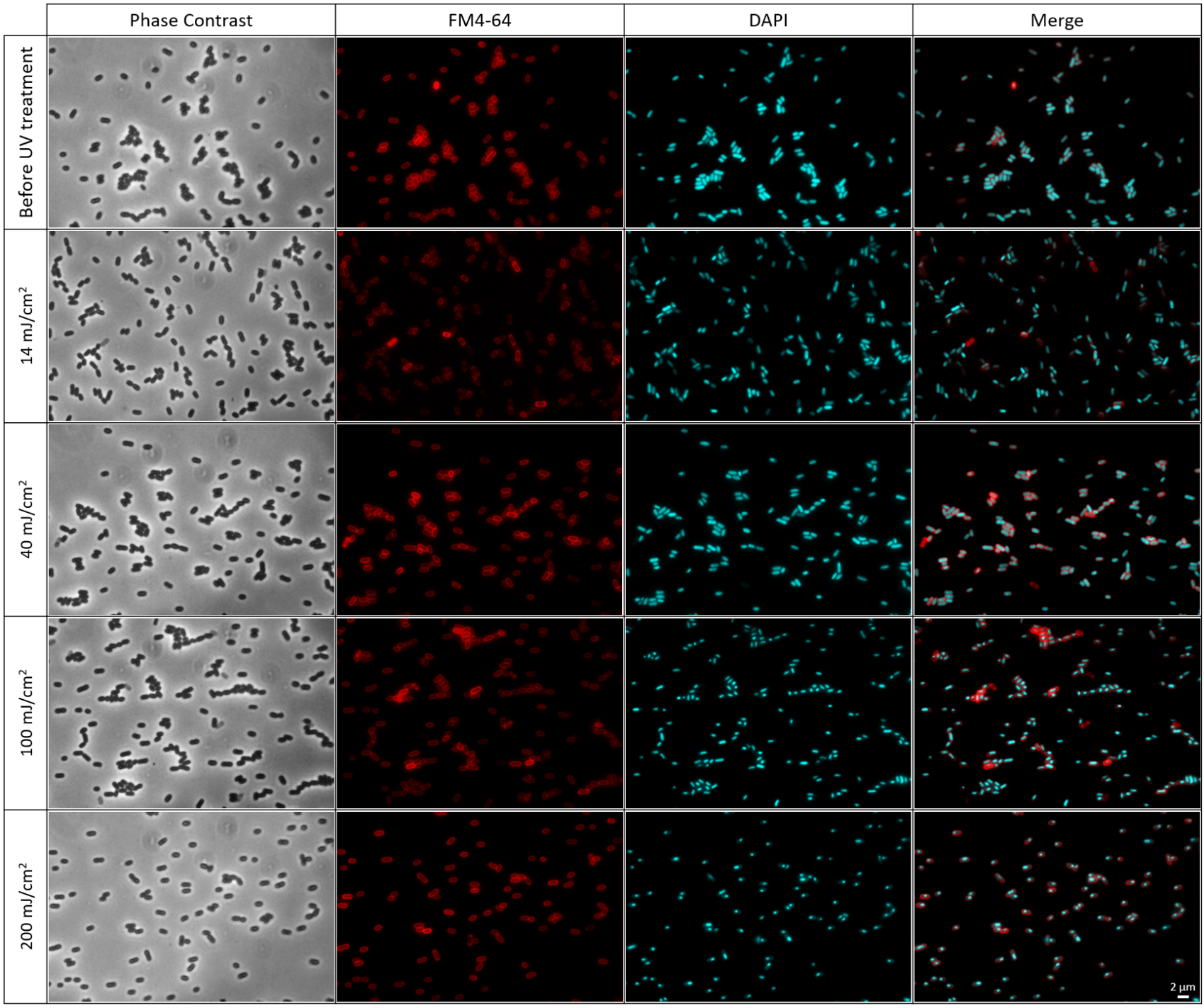


**Figure S13 -** Overview of DAPI-stained *E. coli* nucleoids before and after exposure to 265 nm UV-C LEDs at fluences of 14 mJ/cm², 40 mJ/cm², 100 mJ/cm², and 200 mJ/cm². From the left side, the first column presents phase contrast images (grayscale), the second shows membrane staining with FM4-64 (red), the third displays DNA stained with DAPI (cyan), and the fourth shows the merged images of FM4-64 and DAPI. These images represent wider fields of view corresponding to the close-ups shown in Figure 6.

Supplementary Section Tables

**Table S1 –** Technical parameters and spectral characterization of the LEDs emitting at different wavelengths. Measurements performed using a ILT950-UV radiometer.

|  | 255 nm | 260 nm | 265 nm | 270 nm | 280 nm |
| --- | --- | --- | --- | --- | --- |
| Peak Wavelength (nm) | 254 | 258 | 268 | 267 | 279 |
| Full Width Half Maximum (nm) | 11.6 | 10.3 | 12.1 | 14.0 | 12.5 |
| Radiant Flux (mW) | 15.2 | 33.5 | 25.9 | 44.2 | 81.9 |
| Total Irradiance (µW/cm^2^) | 112 | 359 | 255 | 489 | 788 |
| Total Illuminance (lux) | 0.03 | 0.05 | 0.10 | 0.04 | 0.07 |
| PAR (µmol/m^2^/s) | 0.02 | 0.04 | 0.07 | 0.03 | 0.04 |
| VIS [380-450nm] (W/m^2^) | 0.01 | 0.02 | 0.03 | 0.01 | 0.01 |
| UVA [321-390] (µW/cm^2^) | 0.9 | 1.5 | 1.3 | 1.1 | 1.4 |
| UVB [281-320] (µW/cm^2^) | 0.6 | 3.1 | 12.4 | 22.3 | 386 |
| UVC [<280] (µW/cm^2^) | 110 | 353 | 238 | 464 | 392 |

**Table S2 –** Spectral characterization of the five LEDs used for photoreactivation experiments. The measurement was performed below each LED in the same distance as the samples. Measurements performed using a UPRtek PG100N spectrometer.

|  | LED 1 | LED 2 | LED 3 | LED 4 | LED 5 |
| --- | --- | --- | --- | --- | --- |
| Peak emission ʎp (nm) | 451 | 451 | 450 | 450 | 449 |
| ʎpV (mW/m^-2^) | 146 | 163 | 162 | 153 | 141 |
| PFD-UV [380-400nm] (µmol/(m^2^*s) | 0.08 | 0.09 | 0.08 | 0.07 | 0.06 |
| PPFD [400-700nm] (µmol/(m^2^*s) | 81.2 | 90.5 | 89.6 | 84.1 | 76.7 |
| PFD-FR [700-780nm] (µmol/(m^2^*s) | 2.4 | 2.6 | 2.6 | 2.4 | 2.1 |
| PFD [380-780nm] (µmol/(m^2^*s) | 83.6 | 93.2 | 92.3 | 86.6 | 78.9 |

**Table S3** – Polynomial regression and the correspondent coefficients of determination obtained for Escherichia coli and Enterococcus faecium after inactivation with LEDs emitting at 255 nm, 260 nm, 265 nm, 270 nm and 280 nm.

| **LEDs wavelength (nm)** | **Escherichia coli** | **Enterococcus faecium** |
| --- | --- | --- |
| **255** | y = -0.0731x2 + 1.4815x R² = 0.9082 | y = -0.0522x2 + 1.2074x R² = 0.9196 |
| **260** | y = -0.0702x2 + 1.4052x R² = 0.9174 | y = -0.0627x2 + 1.3816x R² = 0.9381 |
| **265** | y = -0.1177x2 + 2.0978x R² = 0.8561 | y = -0.0639x2 + 1.394x R² = 0.9339 |
| **270** | y = -0.0646x2 + 1.315x R² = 0.8953 | y = -0.0623x2 + 1.379x R² = 0.9451 |
| **280** | y = -0.0472x2 + 1.0445x R² = 0.8564 | y = -0.0598x2 + 1.3609x R² = 0.9515 |

**Table S4 -** Fluence based inactivation rate constants (*kf*) obtained for the culture collection strains of *Escherichia coli and Enterococcus faecium* after exposure to LEDs that emit light at different wavelengths (255, 260, 265, 270, and 280 nm). The high coefficients of determination (R² values in square brackets) ranging from 0.891 to 0.975 indicate a strong fit to the-linear regression, assuring the reliability of the data.

| LEDs wavelenght (nm) | *E. coli* | *E. faecium* |
| --- | --- | --- |
|  | kf (cm^2^/mJ) [R^2^] | k (cm^2^/mJ) [R^2^] |
| 255 | 1.324 [0.947] | 0.902 [0.974] |
| 260 | 1.038 [0.968] | 0.917 [0.945] |
| 265 | 1.393 [0.969] | 0.844 [0.961] |
| 270 | 1.083 [0.891] | 1.264 [0.935] |
| 280 | 1.189 [0.975] | 1.090 [0.975] |

**Table S5 -** Quantification of *E. coli* cells per microscopy image upon exposure to the LEDs emitting light at 265 nm at different UV fluences.

| **UV fluence** | **Sample** | **Total Cells** | **Pi-positive** | **Average PI positive ± standard deviation (%)** |
| --- | --- | --- | --- | --- |
| **0 mJ/cm^2^** | 1 | 102 | 1 | 0.8 ± 0.3 |
|  | 2 | 210 | 1 |  |
|  | 3 | 702 | 6 |  |
| **14 mJ/cm^2^** | 1 | 667 | 15 | 2.9 ± 0.9 |
|  | 2 | 449 | 11 |  |
|  | 3 | 458 | 18 |  |
| **40 mJ/cm^2^** | 1 | 278 | 12 | 5.1 ± 1.3 |
|  | 2 | 152 | 10 |  |
|  | 3 | 324 | 14 |  |
| **100 mJ/cm^2^** | 1 | 233 | 10 | 5.5 ± 1.5 |
|  | 2 | 409 | 21 |  |
|  | 3 | 487 | 35 |  |
| **200 mJ/cm^2^** | 1 | 637 | 37 | 5.7 ± 0.2 |
|  | 2 | 539 | 31 |  |
|  | 3 | 456 | 25 |  |

Supplementary Section Bibliography

[1] Bolton, J. R. & Linden, K. G. Standardization of Methods for Fluence (UV Dose) Determination in Bench-Scale UV Experiments. *Journal of Environmental Engineering* **129**, 209–215 (2003).

[2] Sério, J. *et al*. Wavelength-dependent transcriptomic responses of *Escherichia coli* to UV-C LED irradiation. **Submitted**. 2026

[3] Martín-Sómer, M., Pablos, C., Adán, C., van Grieken, R. & Marugán, J. A review on LED technology in water photodisinfection. *Science of The Total Environment* **885**, 163963 (2023).

[4] Beck, S. E., Wright, H. B., Hargy, T. M., Larason, T. C. & Linden, K. G. Action spectra for validation of pathogen disinfection in medium-pressure ultraviolet (UV) systems. *Water Res* **70**, 27–37 (2015).

[5] Cavaluzzi, M. J. & Borer, P. N. Revised UV extinction coefficients for nucleoside-5’-monophosphates and unpaired DNA and RNA. *Nucleic Acids Res* **32**, (2004).

[6] Li, X. *et al.* Evaluation survey of microbial disinfection methods in UV-LED water treatment systems. *Science of The Total Environment* **659**, 1415–1427 (2019).

[7] Shin, J. Y., Kim, S. J., Kim, D. K. & Kang, D. H. Fundamental Characteristics of Deep-UV Light-Emitting Diodes and Their Application To Control Foodborne Pathogens. *Appl Environ Microbiol* **82**, 2–10 (2015).

[8] Nyangaresi, P. O. *et al.* Comparison of the performance of pulsed and continuous UVC-LED irradiation in the inactivation of bacteria. *Water Res* **157**, 218–227 (2019).

[9] Torkzadeh, H., Zodrow, K. R., Bridges, W. C. & Cates, E. L. Quantification and modeling of the response of surface biofilm growth to continuous low intensity UVC irradiation. *Water Res* **193**, (2021).

[10] Vignaroli, C. *et al.* Epidemic *Escherichia coli* ST131 and *Enterococcus faecium* ST17 in coastal marine sediments from an Italian beach. *Environ Sci Technol* **47**, 13772–13780 (2013).

[11] European Community Commission (ECC), 2006. Directive 2006/7/EC of the European Parliament and of the Council of 15 February 2006 concerning the management of bathing water quality and repealing Directive 76/160/EEC. Off. J. Eur. Union, 64, pp.37–51.
